# Plasma Metabolomics Analysis Based on GC-MS in Infertile Males with Kidney-Yang Deficiency Syndrome

**DOI:** 10.1155/2017/6270195

**Published:** 2017-09-25

**Authors:** Piao Zheng, Yun Wang, Hongmei Lu, Xinyi Zhou, Tao Tang, Rong Fan, Chunhu Zhang, Hanjin Cui, Yang Wang, Jiekun Luo

**Affiliations:** ^1^Department of Integrated Traditional Chinese and Western Medicine, Xiangya Hospital, Central South University, Changsha 410083, China; ^2^College of Chemistry and Chemical Engineering, Central South University, Changsha 410008, China

## Abstract

**Introduction:**

Chinese medicine syndrome diagnosis is the key requisite in the treatment of male infertility with traditional Chinese medicine (TCM). Kidney-Yang deficiency syndrome (KYDS) is the critical Chinese medicine syndrome of male infertility. To explore the modernized mechanisms of KYDS in male infertility, this study aims to investigate the metabolomics of males with KYDS.

**Methods:**

The gas chromatography-mass spectrometry method was applied to analyze the plasma samples of 67 infertile males with KYDS compared with 55 age-matched healthy controls. The chemometric methods including principal component and partial least squares-discriminate analyses were employed to identify the potential biochemical patterns. With the help of the variable importance for the projection and receiver operating characteristic curve analyses, the potential biomarkers were extracted to define the clinical utility. Simultaneously the high-quality KEGG metabolic pathways database was used to identify the related metabolic pathways.

**Results:**

The metabolomics profiles of infertile males with KYDS including 10 potential biomarkers and six metabolic pathways were identified. They precisely distinguished infertile males with KYDS from healthy controls.

**Conclusions:**

These potential biomarkers and pathways suggest the substantial basis of infertile males with KYDS. The metabolomics profiles highlight the modernized mechanisms of infertile males with KYDS.

## 1. Introduction

Infertility is a global health issue affecting 15% of all couples worldwide [1, 2]. Male infertility directly or indirectly contributes to about 60% of infertile couples [3]. Traditional Chinese medicine (TCM) has a long history in the diagnosis and treatment of andropathies including male infertility [4]. The primary cause of male infertility is poor sperm quality. TCM can enhance the sperm motility, attenuate genital inflammatory conditions, and regulate the sexual dysfunction [5]. Particularly, oligospermia treatment with TCM has a better improvement compared with the modern medicine [6].

As far as we know, the correct diagnosis of Chinese medicine syndrome (CMS) mainly contributes to the satisfied effects in the TCM treatment of male infertility. CMS is the basic description of the disease in TCM. According to TCM theory, the imbalance of kidney essence and Qi results in the male infertility [4]. Thus, the tonification of the kidney and promotion of the Qi-blood circulation are used frequently and efficiently to treat male infertility [7]. With regard to the treatment of male infertility, the Kidney-Yang deficiency syndrome (KYDS) is the critical CMS of male infertility [8]. Thus, Jingui Shenqi pills and Yougui pills are the primary formulae to treat male infertility with KYDS [9, 10].

With the development of TCM modernization, the modern connotation of CMS becomes a hot research field [11]. This is because TCM syndrome differentiation always mainly depends on doctors' experience rather than objective index. TCM scientists tend to elucidate the scientific basis of CMS. Fortunately, metabolomics provides a useful tool to explore the essence of CMS and to bridge the gap [9]. Metabolomics reflects the terminal state of a holistic system. It exactly fits the holistic concept of TCM theory. The applications of metabolomics can help to reveal the modernized connotation of CMS and to better understand the potential biological mechanisms [11].

Several studies on traditional Chinese syndrome employed metabolomics method. The sugars and amino acids can help to differentiate between varieties of Qi-Yin Deficiency in prediabetes [12]. The acetyl glutamic acid, lysine, valine, and carnitine have significant differences between Qi Deficiency and non-Qi Deficiency of coronary patients by metabolomics study [13]. Plasma metabolites including acetate, lactate, tyrosine, low density lipoprotein, and unknown compounds (3.44 ppm) can discriminate liver Qi stagnation and spleen deficiency syndrome in rats [14]. Although the metabolomics analysis is widely used in exploring essence of CMS, it has been seldom applied to illuminate the TCM syndromes of male infertility. Precious study has reported seminal metabolomics analysis in male infertility patients with CMS [15]. However, according to TCM theory, traditional Chinese syndrome describes the holistic status of disease. We persist that plasma can reflect the holistic status. Thus, it is essential to investigate the plasma metabolomics in male infertility patients with CMS to complement the data of seminal fluid.

Taken together, this study aims to investigate the changes of metabolites and metabolic pathways from plasma of infertile males with KYDS. Furthermore, we attempt to explore the mechanism of KYDS in male infertility. Plasma metabolomics methods based on gas chromatography-mass spectrometry (GC-MS) and multiple bioinformatics tools were applied to analyze the plasma samples from infertile males with KYDS and healthy controls. This research could help to reveal the substance basis of metabolism of infertile males with KYDS.

## 2. Methods

### 2.1. Participants and Selection Criteria

This study was performed under the guidance of the Helsinki Declaration and approved by the institutional human subjects committee of Xiangya Hospital of Central South University. The number of Ethical Review (Scientific) is 201407389. All participants had written informed consent. All samples were collected from Male Department of Integrated Traditional Chinese and Western Medicine, Xiangya Hospital of Central South University, Changsha, Hunan Province, China. Patients who correspond to inclusion criteria were enrolled in the study. Diagnosis was provided by the same physician.

Inclusion criteria of the experiment group were as follows: (1) males who married with age between 22 and 45 years, (2) couples who could not conceive after 12 months of regular and unprotected intercourse (the male factors cause infertility alone), and (3) males who succeeded to achieve intravaginal ejaculation during intercourse (severe primary diseases in the liver, kidney, endocrine, hematopoietic, or other systems or mental illness were excluded); inclusion criteria were required to correspond to diagnosis standards of KYDS. Standards of KYDS were based on the Chinese Integrated Traditional and Western Medicine Deficiency Syndrome and Geriatrics Research Professional Committee [16]. Primary diagnostic criteria of KYDS were as follows. Primary manifestations included (1) loss of libido, erectile dysfunction, or premature ejaculation, (2) abnormal semen quality such as oligozoospermia (sperm concentration <20 × 10^6^/ml) and asthenozoospermia (less than 50% motile spermatozoa or less than 25% spermatozoa with progressive motility), azoospermia, or teratozoospermia, and (3) weak ejaculation or orgasm disorder. Secondary manifestations included (1) soreness or coldness of the lumbar region or knees, (2) tiredness, accidie, or weakness, (3) increased urine volume or frequency of micturition, (4) pale tongue with a thin and white coating, and (5) deep thready pulse. Patients with at least two primary and two secondary manifestations were diagnosed as KYDS.

Inclusion criteria of the healthy control group were as follows: (1) males who married with age between 22 and 45 years, (2) couples who succeeded to conceive within latest 3 years, (3) males who had the normal semen quality, and their seminal parameters conformed to the WHO 2010 normal reference values (sperm concentration >20 × 10^6^/ml, more than 50% motile spermatozoa, or more than 25% spermatozoa with progressive motility), and (4) males without severe primary diseases in the liver, kidney, endocrine, hematopoietic, or other systems or mental illness.

### 2.2. GC-MS Analysis

Each participating man had a venous blood sample drawn. The plasma was immediately separated (3000 rpm, 15 min, and 25°C) and kept frozen at −80°C for metabolomics analyses. Before GC-MS analysis, 300 *μ*L of methanol was added to each 100 *μ*L plasma sample to precipitate the proteins. At the same time, internal standard was added to sample, which is 30 *μ*L 2-isopropylmalic acid/methanol (1 mg/mL). Then, it was mixed for 15 s and centrifuged at 16000 rpm for 10 min at 4°C to remove proteins. The supernatant (330 *μ*L) was put into a dry tube and evaporated to dryness by nitrogen. 50 *μ*L methoxyamine/pyridine (20 mg/mL) was added and mixed for 30 s, and then the mixture was incubated for 1 hour at 70°C with a plug. 100 *μ*L BSTFA derivatization agents were added, mixed, and incubated. To ensure data quality for metabolic profiling, quality control samples were prepared by pooling and mixing 35 *μ*L aliquots from each plasma sample. Quality control samples were used to assess and ensure that the analysis processes being performed are proper and the data acquired meet predefined acceptance criteria [17]. We tested the stability and repeatability of metabolomic method by the relative standard deviations (RSDs). When examining repeatability of method, the RSDs of peak area for 37 metabolites were within the ranges of 1.010%–21.345%. When examining the stability of the instrument, the RSDs of peak area for 37 metabolites were within the ranges of 3.207%–20.721% (creatinine enol was 49.857%). These results validated the stability and reproducibility of the metabolomic method.

Gas chromatography was depending on a Shimadzu GC-2010 gas chromatography instrument (Shimadzu, Japan). Carrier gas was helium used with a flow rate of 1.0 mL/min. The column was an Agilent DB-5MS (30 m × 0.25 mm, 0.25 *μ*m) with the column temperature maintained at 70°C for 4 min and then heated at a rate of 8°C/min up to 300°C maintained for 3 min. The flow rate was 3 mL/min. Ionization energy was 70 eV. The Shimadzu QP2010 mass spectrometer (Shimadzu, Japan) was operated under electron impact (EI) in a detector voltage of 0.95 kV, scan range of 35 to 800 *m*/*z*, and scan time of 0.2 s.

### 2.3. Data Processing and Analysis

Data pretreatment procedures of each plasma sample, such as retention characteristics, peak intensities, and integrated mass spectra, were performed by NIST Mass Spectral Search Program Version 2.0. Then, the data were reformed into an Excel (Microsoft, Redmond, Washington) matrix in which the rows represent 122 samples and the columns represent 37 metabolites' relative concentrations (%). The matrix was then imported into SIMCA-P software (Version 13.0, Umetrics AB, Umea, Sweden) for multivariate statistical analysis.

To maximize identification of differences in metabolic profiles between groups, principal component analysis (PCA) and partial least squares-discriminate analysis (PLS-DA) were employed. Potential biomarkers were extracted from the values of variable importance plot (VIP) combined with the *P* value of Student's* t*-test. Heat maps and hierarchical cluster analyses were conducted using MeV version 4.9.0. Receiver operating characteristic (ROC) curve analysis was applied in defining clinical utility of a biomarker [18]. Statistical analyses were performed using SPSS 22.0 (SPSS Inc., Chicago, IL, USA). *P* value of < 0.05 was considered statistically significant.

### 2.4. Biological Pathway Analysis

Potential biomarkers were subjected to pathway analysis with MetaboAnalyst 3.0 (http://www.metaboanalyst.ca/) which is a free tool based on the high-quality KEGG (http://www.kegg.jp/kegg/pathway.html) metabolic pathways database to identify related metabolic pathways [19]. The pathway impact value was calculated from pathway topology analysis. Pathways with values higher than 0.1 were screened as potential target pathways and might be used to differentiate infertile males from KYDS from healthy controls.

## 3. Results

### 3.1. Grouping and Data

During August 2014 to December 2014, 122 participants were collected and divided into KYDS group and healthy control (HC) group. KYDS group contained 67 infertile males and HC group included 55 age-matched fertile males. The average age of KYDS group was 29.01 (range from 23 to 45) and HC group was 30.45 (range from 22 to 44). In KYDS group, 24 patients were erectile dysfunction or premature ejaculation, 43 patients were spermatozoan abnormalities which contain 22 oligozoospermia and 14 asthenozoospermia and seven azoospermia or teratozoospermia. For all participants, the relative concentrations of 37 metabolites were obtained and reorganized into an Excel matrix as original data.

### 3.2. Biomarkers Identification

**Figure 1:**
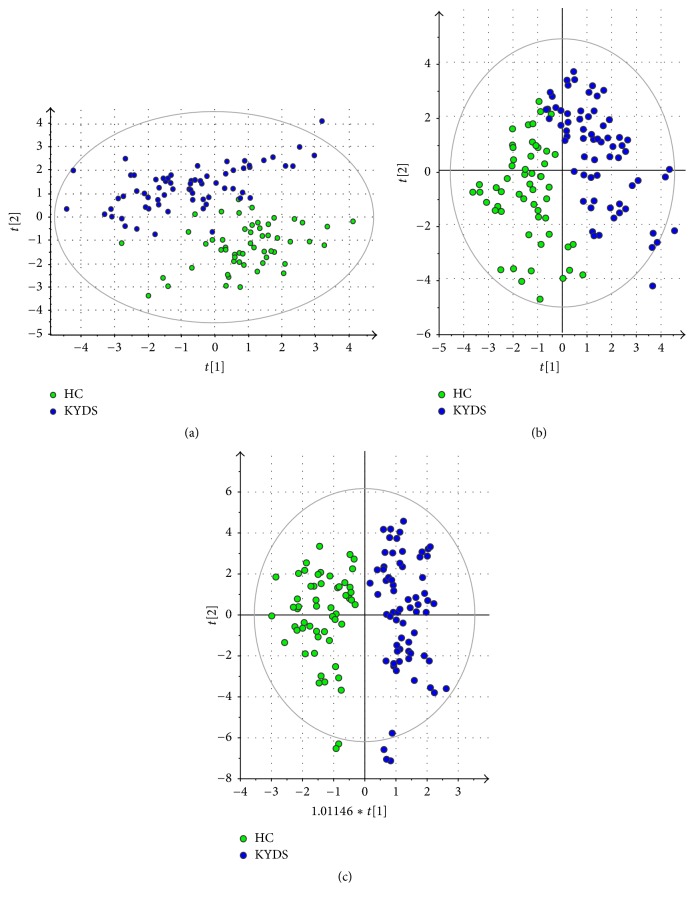
*Score plots of PCA (a), PLS-DA (b), and OPLS-DA (c)*. Each dot represents a plasma sample. Green dots represent the samples of HC group. Blue dots represent the samples of KYDS group.

As an unsupervised multivariate analysis method, the PCA model provided an overview of all plasma samples of 122 participants. In the PCA model, each dot represented a plasma sample; dots with similar metabolomic compositions were clustered together while different metabolic components were dispersed. In Figure 1(a), the distribution of KYDS group and HC group differed obviously, although partially overlapped.

**Figure 2:**
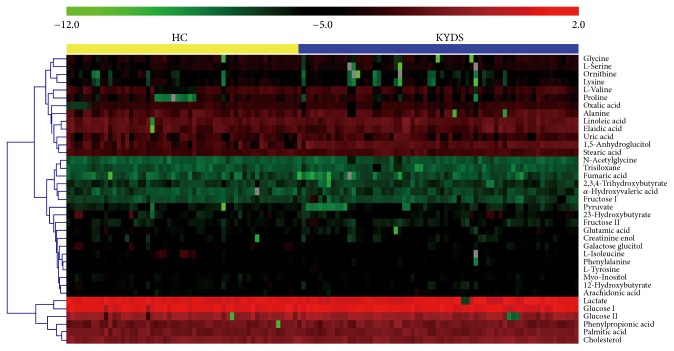
*Heat map of 37 metabolites in the KYDS and HC group.* In the heat map, the rows represent the 37 metabolites and the columns represent samples in the HC and KYDS groups.

Supervised PLS-DA and OPLS-DA analysis techniques were used to search biomarkers. The quality of these models can be explained by *R*^2^ and *Q*^2^ values. The performance characteristics of PLS-DA model were as follows: *R*^2^(*X*) = 0.231, *R*^2^(*Y*) = 0.715, and *Q*^2^(*Y*) = 0.622. Similarly, the performance characteristics of OPLS-DA model were as follows: *R*^2^(*X*) = 0.286, *R*^2^(*Y*) = 0.812, and *Q*^2^(*Y*) = 0.708. These results (Figures 1(b) and 1(c)) demonstrated the existence of different plasma biological signatures between infertile males in the KYDS group and fertile males in the HC group. The OPLS-DA loading plot showed that the scatters of the two groups were completely separated.  Figure 2 was a heat map showing the average normalized quantities of the 37 metabolites in the KYDS and HC group.

**Figure 3:**
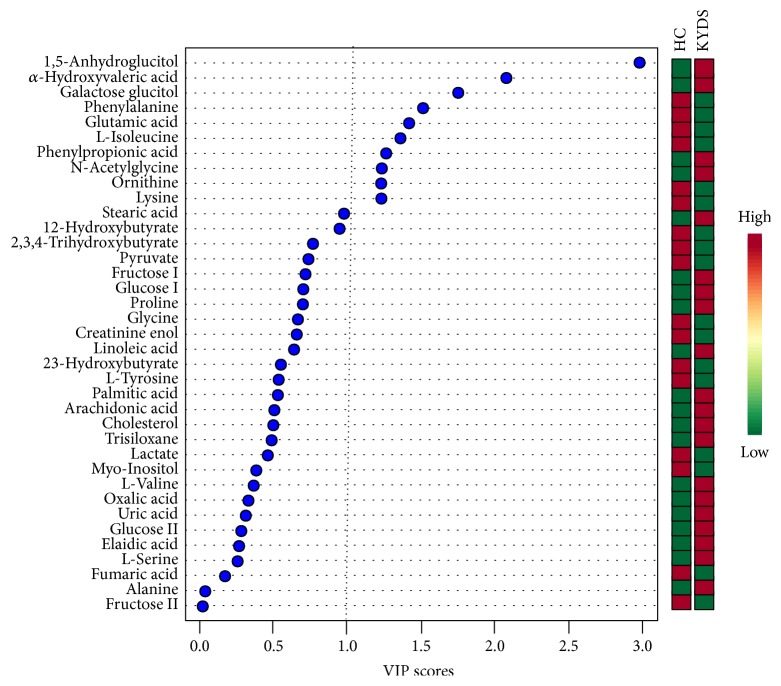
*The VIP plot in OPLS-DA.* The VIP plot and weighted sum of absolute regression coefficients (coef) in OPLS-DA. The VIP plot is sorted from high to low. The colored boxes on the right indicate the relative concentrations of the corresponding metabolite in each group under study. The dash line indicates critical value of VIP values.

A supervised OPLS-DA analysis technique was used to search biomarkers based on the VIP between KYDS and HC group. The VIP values larger than one indicated the importance of the variables.  Figure 3 showed the 37 metabolites sorted from high to low according to the VIP values and their weighted sum of absolute regression coefficients (coef).

**Figure 4:**
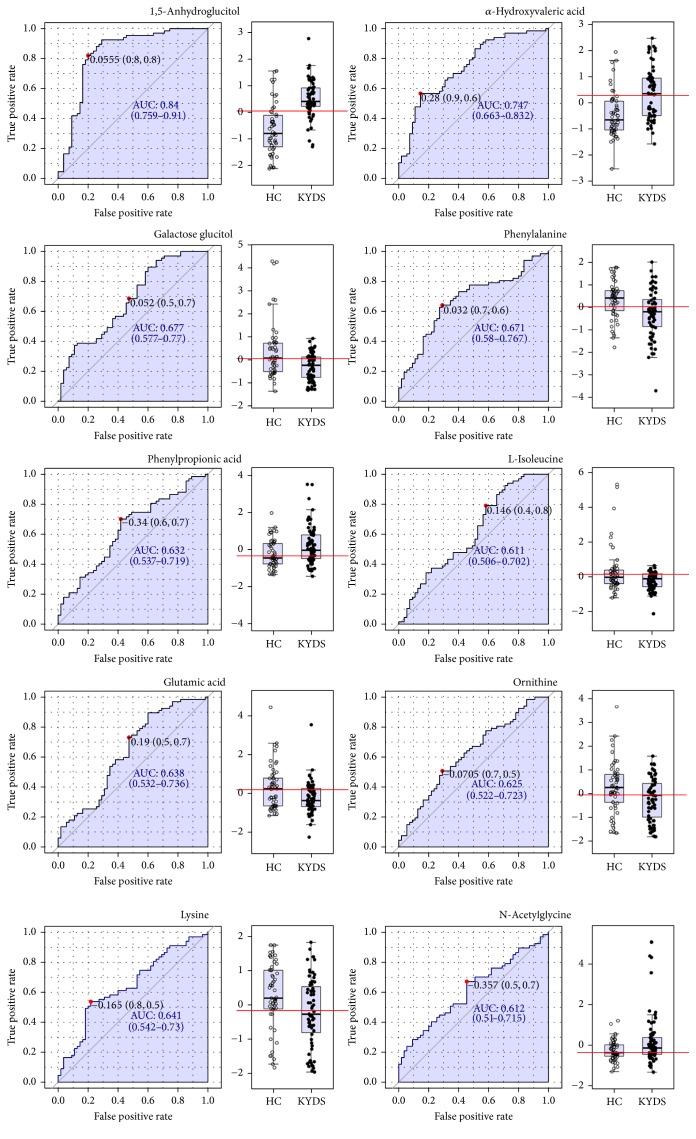
*ROC curve of 10 potential biomarkers*. The ROC curve analyses were based on AUROC of the 10 potential biomarkers. The 95% confidence interval was calculated using 500 boot strappings. The red line shows the cutoff value.

Classical univariate ROC curve analyses were applied in biomarker analyses based on area under ROC curve (AUROC),* P* value, and fold change (FC). The features displayed in Table 1 showed that a total of 10 discriminating metabolites (VIP > 1.0, AUC > 0.6) were identified as biomarkers. Their AUROC were showed in Figure 4. The 10 potential biomarkers are 1,5-anhydroglucitol, *α*-hydroxyvaleric acid, galactose glucitol, phenylalanine, glutamic acid, L-isoleucine, phenylpropionic acid, N-acetylglycine, ornithine, and lysine.

### 3.3. Pathway Analysis

**Figure 5:**
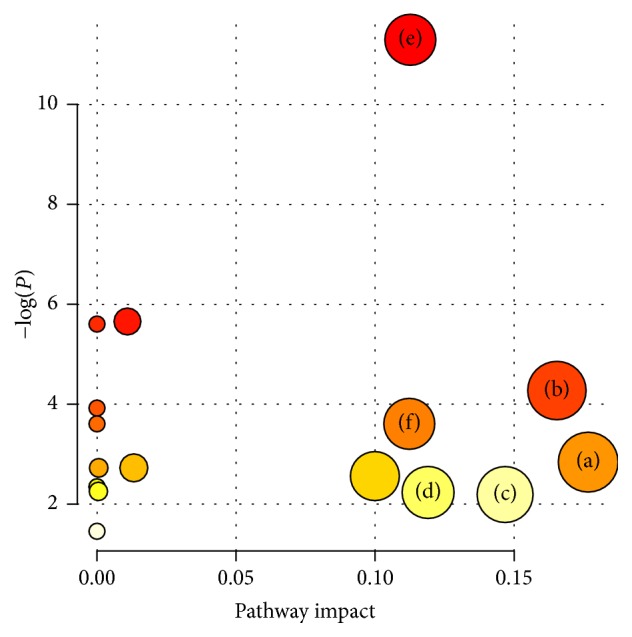
*Summary of pathway analysis.* (a) Alanine, aspartate, and glutamate metabolism. (b) Arginine and proline metabolism. (c) Lysine degradation. (d) Phenylalanine metabolism. (e) Aminoacyl-tRNA biosynthesis. (f) D-Glutamine and D-glutamate metabolism. The node color is based on its *P* value and the node radius is based on their pathway impact values.

**Figure 6:**
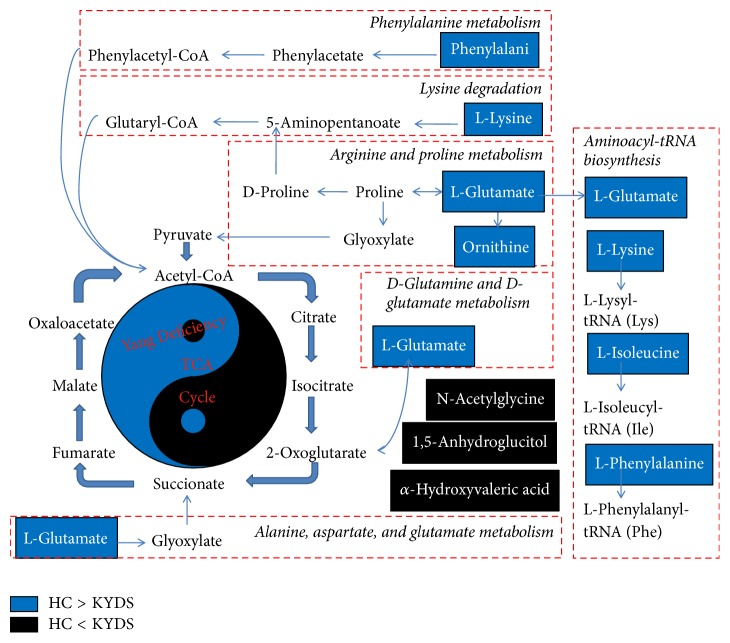
*Detailed construction of the six potential pathways.* The map shows connection of the alanine, aspartate, and glutamate metabolism, arginine and proline metabolism, lysine degradation, phenylalanine metabolism, aminoacyl-tRNA biosynthesis, D-glutamine, and D-glutamate metabolism in human. The map was generated using the reference map by KEGG (http://www.genome.jp/kegg/). The metabolites marked by blue boxes indicate that the weighted sum of absolute regression coefficients in KYDS group is lower than HC group. The metabolites marked by black boxes indicate the reverse results.

MetaboAnalyst 3.0 was used to help identify the most relevant pathways according to the 10 potential biomarkers. The 10 compound labels were standardized (Table 1) before compared with compounds contained in the pathway library. Compounds without match would be excluded from the subsequently pathway analysis. Pathways with values higher than 0.1 were screened as potential target pathways and might be used to differentiate infertile males with KYDS from fertile males with HC. The six potential pathways were alanine, aspartate, and glutamate metabolism, arginine and proline metabolism, lysine degradation, and phenylalanine metabolism, aminoacyl-tRNA biosynthesis, and D-glutamine and D-glutamate metabolism (Figure 5). The detailed results of the pathway analysis were showed in Table 2.  Figure 6 displayed the interrelation of the six potential pathways. The map in Figure 6 was generated using the reference map by KEGG (http://www.genome.jp/kegg/).

## 4. Discussion

In TCM, Chinese medicine syndrome is critical to prescription. As an ancient system, CMS is a subjective diagnosis. It is different from modern medicine. Therefore, we need a sensitive and accurate tool to distinguish TCM syndrome in an objective way. Fortunately, omics method, such as metabolomics, tends to be such a powerful tool for the CMS diagnosis [16]. It makes CMS into modern science. Thus, the present study employed GC-MS based metabolomics detection to elucidate the discrimination between infertile males with KYDS and healthy controls from metabolomics profiles.

Compared with liquid chromatograph-mass spectrometry (LC-MS), GC-MS has larger commercial and public libraries. In addition, GC-MS method keeps the retention times reproducible which LC-MS can not arrive. LC-MS shows the problems of ion suppression and adduct. It has different metabolites detected in positive and negative scanning mode. Moreover, the metabolite identification by LC-MS is difficult and complex because of the lack of comprehensive spectral libraries. Due to the destruction to the sample by LC-MS, reproducibility of retention time between the negative and the positive scanning mode is difficult [20]. Therefore, we used GC-MS method in this study. GC-MS is widely used for the metabolites analyze of human plasma samples. Previous studies have succeeded to apply GC-MS to quantitative analysis of drugs in human plasma [21, 22]. In our study, we chose GC-MS to identify 37 metabolites between KYDS and HC plasma. Chemometric methods including PCA, PLS-DA, and OPLS-DA plots helped to further separate metabolites. OPLS-DA model is the best way to construct for discrimination of KYDS and HC patients. Particularly, it revealed the existence of biomarkers to distinguish between these two types of patients.

Bioinformatics tools are widely applied in finding and identifying biomarkers and metabolic pathways. According to bioinformatics rule, VIP values (VIP > 1) from the OPLS-DA model and *P* value from Student's *t*-test. (*P* < 0.05) are thought to be significantly distinguished [23]. Further, OPLS-DA combined with AUROC is available to find potential biomarkers [24]. In this study, based on OPLS-DA model, we adopted VIP values, AUROC, and Student's *t*-test to screen 10 biomarkers from 37 metabolites. Furthermore, we accurately used MetaboAnalyst 3.0 platform to help identify the most related six metabolic pathways. With the help of biological information database (KEGG), we mapped the metabolic pathways in KYDS and HC biomarkers profiles.

Previous study reported the metabolomics detection of infertile males with KYDS using seminal plasma [15]. They found that 41 metabolites were related to infertile males with KYDS, and seven metabolites were related to the five potential metabolic pathways [15]. However, we think that the results of seminal plasma ca not describe the metabolomics profiles of infertile males with KYDS. According to TCM theory, traditional Chinese syndrome describes the holistic status of disease. We persist that plasma can reflect the holistic status. Thus, in the present research, we analyze the plasma of infertile males with KYDS by GC-MS based metabolomics method compared with HC. Ultimately, 10 potential biomarkers were identified; further six relative metabolic pathways were anchored. Compared with the seminal plasma study, only phenylalanine metabolism pathway was the same. The other discriminate metabolism pathways in our study were different from the seminal plasma study. This difference may be due to the patients group, specimen source, metabolite method, and database. It is manifested that plasma metabolomics is differ from seminal plasma metabolomics in male infertility patients with CMS. Our research is essential and can complement the data of seminal plasma.

Asthenozoospermia is more prevalent and important than oligozoospermia in infertile males with KYDS [15]. In our study, 24 patients with erectile dysfunction or premature ejaculation, 22 with oligozoospermia, and 14 with asthenozoospermia in the infertile males of KYDS were observed. According to the results, erectile dysfunction or premature ejaculation is the potential important type in infertile males with KYDS.

This study found 10 potential biomarkers (1,5-anhydroglucitol, *α*-hydroxyvaleric acid, galactose glucitol, phenylalanine, glutamic acid, L-isoleucine, phenylpropionic acid, N-acetylglycine, ornithine, and lysine) and six metabolic pathways (alanine, aspartate, and glutamate metabolism, arginine and proline metabolism, lysine degradation, phenylalanine metabolism, aminoacyl-tRNA biosynthesis, D-glutamine, and D-glutamate metabolism) can be used to discriminate infertile males with KYDS from healthy controls. Among these 10 potential biomarkers, 1,5-anhydroglucitol is the most important metabolite. It can be used to discriminate glucose variability in diabetics [25]. Diabetes is closely associated with male infertility [26]. Diabetes can disrupt the formation of sperm, penile erection, and ejaculation. Thus, 1,5-anhydroglucitol can influence male reproductive function. Diabetics were not included in this study; we can speculate that infertile males with KYDS might be with saccharide utilization disorders. *α*-Hydroxyvaleric acid is the second important metabolite. The oxidative stress markers in semen cause sperm damage and sperm malformation, resulting in the male infertility [27–29]. Oxidative stress in plasma may be caused by KYDS in male infertility. The principle of changes is not clear and need to be further studied.

Moreover, we found six lower amino acid metabolites including galactose glucitol, phenylalanine, glutamic acid, L-isoleucine, ornithine, and lysine content of infertile males with KYDS compared with HC (Figure 3). This phenomenon indicates that the consumption of amino acids compounds exists in KYDS. This is the major character in biomarkers we found. Symptoms of fatigue, lethargy, or weakness in KYDS might be relative to these amino acids compounds consumption.

The metabolic pathways profiles show that the six metabolic pathways with higher impact are implied in TCA cycle (Figure 6). This result is consistent with the previous study [30]. Lysine acetylation associated with the level of lysine can impact sperm capacitation [31, 32]. Phenylalanine is transported into tyrosine by phenylalanine hydroxylase. This process may cause the phenylalanine metabolism [33]. As a result, the deficiency of the functional protein acetylation and the activity of phenylalanine hydroxylase finally influence the TCA cycle. We can suggest that the KYDS of male infertility may refer to this process.

TCA cycle is closely connected with energy metabolism. The biomarkers we found are the great mass of amino acids. It is possible that infertile males with KYDS are associated with energy consumption and antioxidant defenses. Oligozoospermia may be tightly associated with energy consumption and antioxidant defenses in spermatogenesis [10]. KYDS is relevant to the disorders of energy and amino acid metabolisms [34]. These studies support our result. Morinda can improve energy metabolism and antioxidant defenses in KYDS [35]. It proved that Chinese herbs could reverse these effects.

Although GC-MS was applied to analyze samples of KYDS of infertility, more analysis methods should be used. We should combine LC-MS and NMR to detect more metabolites. Furthermore, multiple centers and more patients should be enrolled in the future study. Finally, the potential biomarkers we found are needed to be further clinically validated to help the clinical diagnosis of infertile males with KYDS.

## 5. Conclusion

In summary, the metabolomics profiles of infertile males with KYDS including 10 potential biomarkers and six metabolic pathways were identified. They precisely distinguished infertile males with KYDS from the healthy controls. These potential biomarkers and pathways suggest the substantial basis of infertile males with KYDS. The metabolomics profiles highlight the modernized mechanisms of infertile males with KYDS.

## Supplementary Material

S1 File: Original data of all participants. The rows represent 122 samples and the columns represent 37 metabolites' relative concentrations.
S2 File: Ethics Review Table.
S3 File: User's Guide of NIST program.
S4 File: Tables.

## Figures and Tables

**Table 1:** Results of ROC curve analyses.

Var ID (Primary)	VIP	*P* value	log2 FC	HMDB	PubChem	KEGG
1,5-Anhydroglucitol	2.982	2.94*E* − 12	−0.7639	HMDB02712	64960	C07326
*α*-Hydroxyvaleric acid	2.0807	4.00*E* − 06	−0.34771	HMDB00531	107802	NA
Galactose glucitol	1.7538	1.25*E* − 04	0.34964	NA	NA	NA
Phenylalanine	1.5157	0.0010083	0.24256	HMDB00159	6140	C00079
Glutamic acid	1.4221	0.0020929	0.3106	HMDB00148	33032	C00025
L-isoleucine	1.3639	0.0032191	0.27356	HMDB00172	6306	C00407
Phenylpropionic acid	1.266	0.0063839	−0.27209	NA	NA	NA
N-Acetylglycine	1.2364	0.0077752	−0.13658	HMDB00532	10972	NA
Ornithine	1.2325	0.0079736	0.42849	HMDB00214	6262	C00077
Lysine	1.2321	0.0079989	0.46648	HMDB00182	5962	C00047

The first four columns show the Var ID (primary), *P* value, FC, and VIP values of the 10 biomarkers. The last three columns show the conversion results in MetaboAnalyst 3.0. NA indicates no match. The table ranked by VIP values.

**Table 2:** The detailed results from the pathway analysis.

Pathway name	Hits/Total	Expected	Raw *p*	−log⁡10(*P*)	Holm adjust	FDR	Impact
Alanine, aspartate, and glutamate metabolism	1/24	0.059826	0.058413	2.8402	1	0.52408	0.17664
Arginine and proline metabolism	2/77	0.19194	0.01394	4.273	1	0.2788	0.16538
Lysine degradation	1/47	0.11716	0.1117	2.192	1	0.55848	0.14675
Phenylalanine metabolism	1/45	0.11217	0.10717	2.2334	1	0.55848	0.11906
Aminoacyl-tRNA biosynthesis	4/75	0.18695	1.25*E* − 05	11.293	0.000997	0.000997	0.11268
D-Glutamine and D-glutamate metabolism	1/11	0.02742	0.027137	3.6069	1	0.31013	0.1123
Lysine biosynthesis	1/32	0.079767	0.07724	2.5608	1	0.55848	0.09993
Valine, leucine, and isoleucine biosynthesis	1/27	0.067304	0.06551	2.7255	1	0.52408	0.01325
Glutathione metabolism	2/38	0.094724	0.003499	5.6554	0.2764	0.098232	0.01095
Phenylalanine, tyrosine, and tryptophan biosynthesis	1/27	0.067304	0.06551	2.7255	1	0.52408	0.00062
Histidine metabolism	1/44	0.10968	0.10489	2.2548	1	0.55848	0.00051
Nitrogen metabolism	2/39	0.097216	0.003684	5.6038	0.28733	0.098232	0
D-Arginine and D-ornithine metabolism	1/8	0.019942	0.019797	3.9222	1	0.31013	0
Biotin metabolism	1/11	0.02742	0.027137	3.6069	1	0.31013	0
Valine, leucine, and isoleucine degradation	1/40	0.099709	0.095753	2.346	1	0.55848	0
Butanoate metabolism	1/40	0.099709	0.095753	2.346	1	0.55848	0
Porphyrin and chlorophyll metabolism	1/104	0.25924	0.23302	1.4566	1	1	0

In the table, the “Total” is the total number of compounds in the pathway, the “Hits” is the actually matched number from the use uploaded data, the “Raw *p*” is the original *P* value calculated from the enrichment analysis, the “Holm *p*” is the *P* value adjusted by Holm-Bonferroni method, the “FDR *p*” is the *P* value adjusted using False Discovery Rate, and the “Impact” is the pathway impact value calculated from pathway topology analysis.

## Abbreviations

TCM: Traditional Chinese medicine
CMS: Chinese medicine syndrome
KYDS: Kidney-Yang deficiency syndrome
GC-MS: Gas chromatography-mass spectrometry
LC-MS: Liquid chromatograph-mass spectrometry
PCA: Principal component analysis
PLS-DA: Partial least squares-discriminate analysis
OPLS-DA: Orthogonal partial least squares-discriminate analysis
VIP: Variable importance plot
ROC: Receiver operating characteristic.
