# Supplementary material for: Plasma Metabolomics Analysis Based on GC-MS in Infertile Males with Kidney-Yang Deficiency Syndrome
Source: Evid Based Complement Alternat Med. 2017 Sep 25;2017:6270195. doi: 10.1155/2017/6270195 (PMC5674502; doi:10.1155/2017/6270195)
Supplement: Supplementary file 1 — S1 File: Original data of all participants. The rows represent 122 samples and the columns represent 37 metabolites' relative concentrations. S2 File: Ethics Review Table. S3 File: User's Guide of NIST program. S4 File: Tables. [file 6270195.f1.zip › S2 File. Ethics Review Table (1).pdf]

Scientific Research Project Review and Approval of Medical Ethics Committee of the Xiangya Hospital of Centre South University

Ethical Review (Scientific) No. 201407389

Project Name	Plasma Metabolomics Analysis based on GC-MS in Infertile Males with Kidney-Yang Deficiency Syndrome				
Specialty	Andrology of TCM	Responsibility	Principal ☑<br>Participant ☐	Principal Investigator	Jiekun Luo
Category	1☑ 2☐ 3 ☐ 4☐ 5☐		Research Time	August 2014 to December 2014	
Type	Clinical Research				
Research Institution	Male Department of Integrated Traditional Chinese and Western Medicine, Xiangya Hospital of Central South University				
Comment	Consent■;Need Modification☐;Disagree☐;Termination☐; Pause the experiment☐				

The main content of the research involving human research ethics:

The research needs a venous blood sample drawn from each participant. Plasma samples are collected for metabolomic analyses based on GC-MS. No drug intervention. All participants had written informed consent. The research content and results do not involve participant's personal privacy; there is no conflict of interest.

Ethical review comment:

The research was reviewed and approved by the Ethic Committee of the Xiangya Hospital of Central South University. This is to certificate that the research design and methods are in accordance with the requirements of regulations and procedures regarding to human subject protection laws such as GCP and ICH-GCP.

Notes: Research Category: 1 = The use of pathological tissue; 2 = Human trial; 3 = Human tissue or cell test; 4 = Embryonic cloning research; 5 = Others.
Research Type: 1.Clinical Research; 2.Fundamental Research

Ethic Committee of the
Xiangya Hospital of Central South University
Date of review and approval: 2014.07.10

医学伦理委员会
