# Supplementary material for: Plasma Metabolomics Analysis Based on GC-MS in Infertile Males with Kidney-Yang Deficiency Syndrome
Source: Evid Based Complement Alternat Med. 2017 Sep 25;2017:6270195. doi: 10.1155/2017/6270195 (PMC5674502; doi:10.1155/2017/6270195)
Supplement: Supplementary file 1 — S1 File: Original data of all participants. The rows represent 122 samples and the columns represent 37 metabolites' relative concentrations. S2 File: Ethics Review Table. S3 File: User's Guide of NIST program. S4 File: Tables. [file 6270195.f1.zip › S3 File. User Guide of NIST program (1).pdf]

NIST Standard Reference Database 1A

NIST/EPA/NIH Mass Spectral Library (NIST 08) and NIST Mass Spectral Search Program (Version 2.0f)

For Use with Microsoft® Windows

User's Guide

The NIST Mass Spectrometry Data Center

Stephen E. Stein, Director

Evaluators:

Anzor Mikaia, Principal
Edward White V (EI)
Vladimir Zaikin (EI)
Damo Zhu (EI)
Boris Milman (MS/MS)
Valeri Babushok (RI)
Igor Zenkevich (RI)

Programmers:

Peter Linstrom
Yuri Mirokhin
Dmitrii Tchekhovskoi

Documentation and Help:

W. Gary Mallard
O. David Sparkman
Joan A. Sparkman

June 2008

U.S. Department of Commerce

National Institute of Standards and Technology
Standard Reference Data Program
Gaithersburg, MD 20899

The National Institute of Standards and Technology (NIST) uses its best efforts to deliver a high-quality copy of the Database and to verify that the data contained therein have been selected on the basis of sound scientific judgment. However, NIST makes no warranties to that effect; and NIST shall not be liable for any damage that may result from errors or omissions in the Database.

© 1987, 1988, 1990, 1992, 1994, 1995, 1996, 1997, 1998, 1999, 2000, 2001, 2002, 2005, 2008 copyright by the U.S. Secretary of Commerce on behalf of the United States of America. All rights reserved.

No part of this Database may be reproduced, stored in a retrieval system, or transmitted, in any form or by any means, electronic, mechanical, photocopying, recording, or otherwise, without the prior written permission of the distributor. Portions of this program are also copyrighted by Microsoft Corp., XVT Software, Inc., and © 1984–1996 FairCom Corporation.

.

Microsoft is a registered trademark of Microsoft Corporation, and Windows is a trademark of Microsoft Corporation. ISIS is a trademark of MDL Information Systems, Inc. "FairCom" and "c-tree Plus" are trademarks of FairCom Corporation and are registered in the United States and other countries. All Rights Reserved.

Certain trade names and company products are mentioned in the text to specify adequately the computer products and equipment needed to use this software. In no case does such identification imply endorsement by the National Institute of Standards and Technology of these computer products and equipment, nor does it imply that the products are necessary the best available for the purpose

Contents

Contents ... 3
Installation ... 4
Introduction ... 5
New Features in NIST 08 and the MS Search Program ... 6
Version 2.0f of the MS Search Program ... 6
NIST 08 ... 6
Retention Indices ... 6
The NIST MS/MS Library ... 7
Tab Displays ... 9
The Lib. Search Tab ... 12
Title Bar, Tool Bar, and Status Bar ... 14
Neutral Loss Display ... 15
Spectral Analysis Utilities for NIST 08 ... 17
Automated Mass Spectrometry Deconvolution and Identification System (AMDIS) ... 17
MS Interpreter ... 17
Example Searches ... 19
User Spectrum and Structure Searches ... 19
Library Search Options ... 20
Automations ... 22
Limits ... 22
Constraints ... 22
The Search Menu ... 23
Search by Any Peaks ... 23
Search by Name ... 24
Sequential Method ... 24
Basis for Interpretation of the Library Search Results ... 25
Substructure Information ... 26
Use with Instrument Data Systems ... 28
Use with Third-Party Drawing Programs ... 30
User Libraries ... 31
NIST Text Format of Individual Spectra ... 32
APPENDIX 1: Creating AUTOIMP Files Used in NIST MS Search V.2.0 ... 33
APPENDIX 2: Using the NIST MS Search Program with ChemStation ... 34
APPENDIX 3: Copy ChemStation and Other Libraries to NIST User Library Format ... 37
APPENDIX 4: Using the NIST MS Search Program with Thermo Electron Corpora ration Xcalibur Software ... 40
APPENDIX 5: Search Algorithms ... 41
Screening ... 41
Search ... 41
APPENDIX 6: Syntax for Some Constraints in Searches ... 43
Syntax of Name Fragments Constraint ... 43
Syntax of Tags in Comment Constraint ... 43
APPENDIX 7: Contributing EI Mass Spectra to the NIST/EPA/NIH Mass Spectral Library ... 47
APPENDIX 8: Support Contacts ... 49

Installation

Windows NT 4.0, Windows 2000, Windows XP, Windows Vista

- Select the **Start** button on the lower left of your screen. Then select **Run** from the **Start** menu.
- Assuming your CD-ROM drive is D, type D:\SETUP.EXE and select **OK**. If your CD-ROM drive is a device other than D, replace D with the appropriate drive letter.
- Follow the instructions presented by the SETUP program.
- In upgrading from previous Windows versions, 1.6-1.7, of the NIST MS Search Program, v.2.0 will be added to the previous version's directory unless the install directory is changed from the default. Previous version of the NIST MS Search Program will remain usable unless the contents of the Database directory is replaced.
- The use of the NIST MS Search Program v.2.0 Help files with Windows Vista requires that WinHlp32 be downloaded from Microsoft and installed.

Introduction

The National Institute of Standards and Technology (NIST) introduced a version of its search software used with the NIST/U.S. Environmental Protection Agency (EPA)/National Institutes of Health (NIH) Mass Spectral Database for Microsoft Windows in 1995. At that time, the NIST Mass Spectral Search Program for Windows (the Program) would allow for various types of comparisons of an acquired or unknown spectrum with the NIST/EPA/NIH Database, its Library of Replicate Spectra, and user libraries. The NIST/EPA/NIH Database was also searchable by multiple names that were associated with various spectra. This Incremental name search, which first appeared in a DOS version of the Program, was what analysts had been looking for since commercial libraries became available with mass spectrometry data systems; “how to retrieve a spectrum of a compound without having to search an acquired spectrum against the Library.” Another unique feature of the Program’s spectral comparison algorithms, even in the DOS version, was that the spectra selected from the library during the presearch had all of their peaks compared with all of the peaks in the sample spectrum. All other commercial library search systems at that time still used condensed spectra.

Other features retained in v.2.0 of the Program that were in the original DOS and Windows versions are the ability to retrieve spectra using **Molecular weight**, **Formula**, **Chemical Abstract Services registry numbers** (**CAS rn**), and **Library Id number**. All these search methods, along with the versatile **Any Peaks** search, are available for use with the NIST/EPA/NIH MS Library and user libraries. In addition, multiple constraints can be added to searches that would limit the searched population. The first Windows version introduced the ability to search multiple libraries simultaneously.

Since the introduction of the first Windows version of the Program, other features have been added: 1) mass defect correction of m/z values in imported spectra, 2) the ability to associate structures as MOL files with user library spectra, 3) the ability to have synonyms associated with spectra in user libraries, 4) the ability to use the **Incremental name** search with user libraries, 5) the ability to evaluate a Hit List from a Neutral Loss or Hybrid Neutral loss ”Similarity” search to determine the probability of the presence and absence of various structures, 6) the ability to estimate the molecular weight of an unknown along with the ability to determine the number of atoms of chlorine and/or bromine that may be present in an analyte that produced a mass spectrum, 7) the addition of the mass spectral interpretation tools **Isoform** and **MS Interpreter**; and, most recently, an MS/MS Search, the ability to estimate retention indexes for most of compounds in the Mass Spectral Library, and the ability to view the spectra and other information in the MS/MS Library that is a part of NIST 08. Since the introduction of the first Windows version of the Program, four new versions of the NIST/EPA/NIH Mass Spectral Library (NIST 98, NIST 02, NIST 05, and NIST 08) have been introduced.

New Features in NIST 08 and the MS Search Program

Version 2.0f of the MS Search Program

The basic software of the NIST MS Search Program for Windows **Version 2.0** is unchanged. A number of new features have been added.

- A new spectrum search for MS/MS spectra in MS/MS libraries, including the NIST MS/MS library.
- Five new Constraints have been added; Tags in Comment, Peptide Sequences, Peptide Mobile Protons, Peptide Charge, and Peptide Number of Residues. Tags in comment, for example, allows searching for the name of the contributor in the EI library.
- A search for information in the Contributor/Comment field which may be particularly useful for certain types of information relevant to MS/MS.
- Flexibility has been added to searches using a Name Fragment in Constraints. It is now possible to specify the presence or absence of any arbitrary part or parts of a name.
- The program does not use or require the installation of NIST fonts.

NIST 08

The 2008 version of the NIST/EPA/NIH Mass Spectral Library (NIST 08) contains four libraries and a database of retention index values. There are three EI libraries, mainlib, replib, and nist_salts. Together they contain 220,460 spectra of 192,108 different chemical compounds. This is an increase of about 29,600 compounds (~18%). The EI Salt library contains 717 spectra of 672 compounds of low volatility not expected to be measurable by GC/MS. Limited annotation has been added indicating if a monomeric molecular ion is observed and the identities of polymeric species. The MS/MS library contains 14,802 spectra of 3,898 positive ions and 1,410 negative ions. The features of this library are described in more detail below.

Contents of the NIST/EPA/NIH (NIST 08) Mass Spectral Library		
Library Name	Library Description	Library Contents
mainlib	Main EI MS library	191,436 spectra
replib	Replicate spectra	28,307 spectra
nist_salts	EI Salt Library	717 spectra
nist_msms	MS/MS Library	14,802 spectra of 3,898 positive and 1,410 negative ions
nist_ri	Retention Index Library	293,247 Kovats RI values for 44,008 compounds

Retention Indices

One of the items in the Text Info tab under Properties is **Retention Indices**. Having a check in the box next to **Retention Index** will cause the experimental data for the retention index to be displayed with the spectra. The number of retention indices displayed is controlled by selecting All or First and entering the number of values to be displayed. The default is to display the first two values. Displaying all values will cause the display to be rather slow in cases where there are a very large number of literature values. There are literature data for 21,940 compounds that are in the **mainlib** Library, and a total of 237,206 annotated Kovats Retention Index values, including literature source and measurement conditions. In addition, for many compounds in the database an **Estimated Kovats Retention Index (RI)** will be displayed based on the structure associated with the spectrum. These values are reported in index units **(iu)** with a confidence interval, e.g. “Confidence interval (Ketones): 57(50%) 246(95%) iu” reported for acetone. This means that there is a 50% probability true value falls within 57 iu

of the estimated value and 95% of the time the true value falls within 246 iu of the estimated value. The analysis is based on error measurements of all the data for the class of the compound given; i.e. in this example, ketones. Provided the text pane view has been set to display Retention Indices, these data will be displayed with the results of any search.

The NIST MS/MS Library

MS/MS spectra, along with an increased number of fields relevant to MS/MS data, are provided in a set of files separate from the Main and Replicates data files that comprise the Library of EI spectra. The MS/MS spectra have been provided by contributors, measured at NIST, and extracted from the literature. In view of the relatively small size of the library, it is intended primarily to serve as a "starter set". It also documents spectrum variations between instrument classes under different conditions. The preparation of the MS/MS Library revealed that at sufficiently high signal-to-noise measurement conditions, modern instruments are capable of providing very reproducible "library-searchable" spectra. Although collision energy can be an important variable, spectra vary in an understandable way depending on the compound and instrument type as well as collision-energy conditions.

The **MSMS tab** allows access to the spectra in the NIST_MSMS Library. Each time this tab is selected, the three panes of the display are blank with the exception of a single entry in the ion list pane, which is on the left of the tab's display. This entry is the **MS/MS Library**. There is a box on the left that has a plus sign (+). Placing the Mouse pointer on this box and clicking expands to the first two levels (Positive Ions and Negative Ions). There are plus boxes next to both Positive Ion and Negative Ion. Clicking on one of these plus boxes results in the display of the next level. This is a list of molecular weights of analytes for which a spectrum was obtained by MS/MS. Clicking on the plus box next to one of these molecular weight listings results in the display of a list of elemental compositions that will also have plus boxes next to them. Click on one of the elemental composition check boxes will result in a display of a list of the compound names that have that elemental composition. In most cases, this is only a single compound. Clicking on the plus box next to the compound name will result in the display of a list of the spectra acquired under MS/MS conditions for this compound. If more than one spectrum was acquired, there is a separate entry for each spectrum.

The listing of each spectrum begins with **ms1** or **ms2**. An **ms1** spectrum is composed of ions arising directly from the ion source region. They can arise from electrospray ionization (ESI) or atmospheric pressure chemical ionization (APCI). These ions are not subjected to collision-induced dissociation after exiting the ion source.

For beam-type instruments (e.g., triple quadrupoles and Q-tof instruments), the **ms2** designation refers to spectra generated by dissociation in a collision cell of a selected precursor ion. For ion trap instruments, the **ms2** designation refers to dissociation in the trap after following the isolation of the precursor ion.

In the case of data acquired using an ion tap mass spectrometer, the **ms1** may have a plus box next to it. Clicking on this plus box results in a subsequent spectrum listed as **ms2**, which may also have a plus box. Selection of the plus box next to the **ms2** spectrum can result in an **ms3** spectrum, which is obtained by the isolation of a product ion as a precursor ion for a subsequent iteration of MS/MS.

Clicking on the **ms2** results in the display of the spectrum as seen in Figure 1. The **ms2** designation is followed with the precursor ion *m/z* value and the collision energy for beam-type instruments.

As can be seen from Figure 1 and Figure 2 on the following page, there is a great deal more information provided in a text format for the spectra in the NIST_MSMS Library.

The MS/MS Library can be searched with any of the **Other Searches** or the Incremental Name Search. All that is necessary is to include the nist_msms Library in the **Include Libs** column of the Search dialog box for the **Other Searches** or as the single library to be searched in the **Incremental Name Search**. The Unknown Spectrum Search and Structure Similarity Search are also applicable. However, the user must understand that MS/MS spectra in general are different from EI spectra as well as the fact that structures in the MS/MS Library refer to the neutral form of the precursor ions.

In absence of **nist_msms** library, the MSMS tab is not displayed.

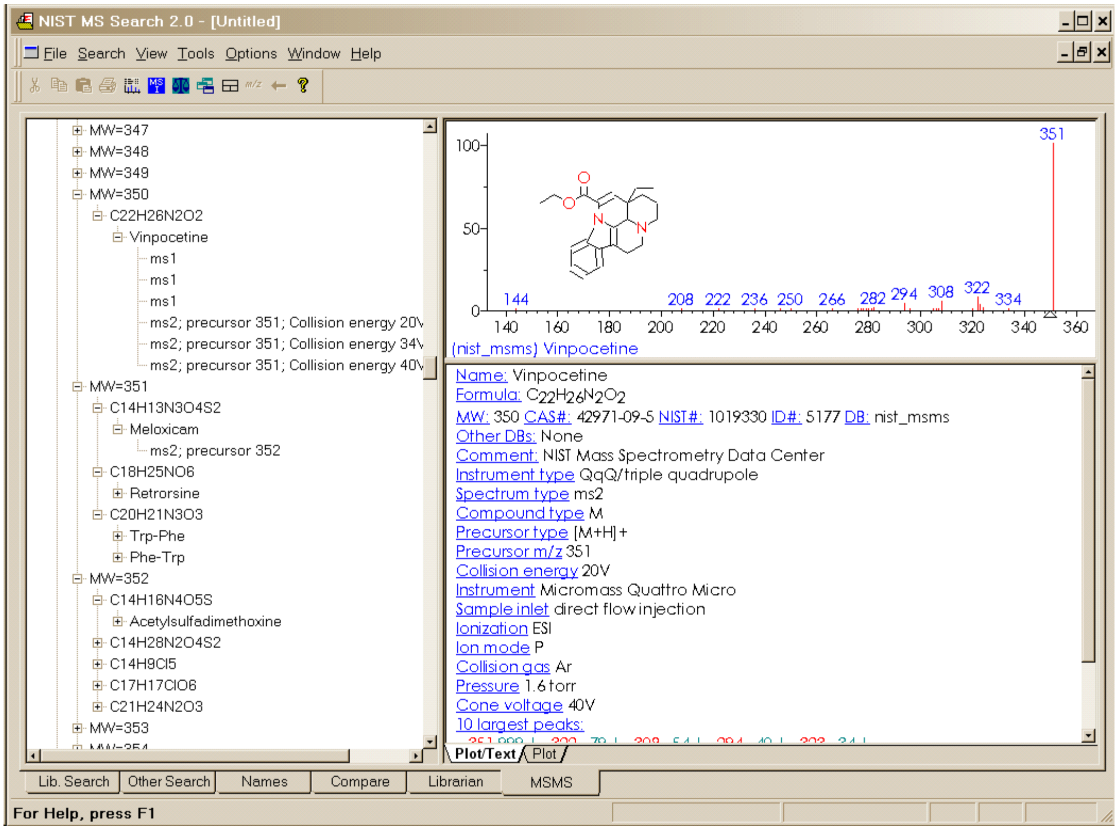

Figure 1. MSMS tab for data acquired using "triple quad".

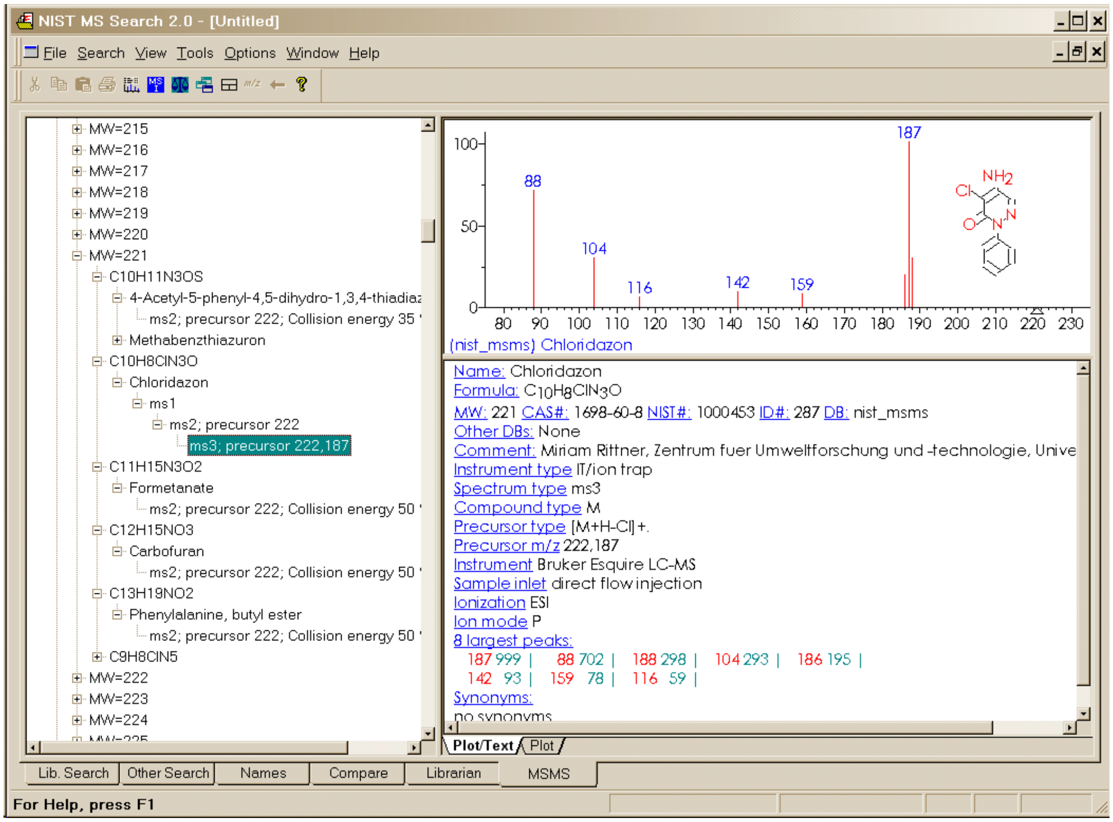

Figure 2. MSMS tab for data acquired using ion trap MS^3.

Tab Displays

Version 2.0 of the Program uses tab displays. Selecting a tab results in the display of a different Window. Each Window serves a different function, and each Window has a number of panes or sections ranging from 5 for the Lib. Search tab to 3 for the Other (searches), Name (incremental name search), Librarian, and MSMS. The panes that are used for spectral displays also have tabs that allow for different types of displays; e.g., a bar-graph spectrum or a bar-graph and text display of the spectrum. These two displays are separated by a splitter bar. The splitter bars allow for the adjustment of the amount of the display designated for the bar-graph spectrum and the text information. The panes on the left side of each Window are reserved for a list of spectra. The pane on the top left of the Lib. Search tab is used for the Spec List, and the pane on the lower left of this Window is used for the Hit List. The pane on the left of the Other and Names tabs is used for the Hit List, and the pane on the Librarian and MSMS tabs is a list of spectra that can be displayed. It is important to know that the Spec List in the Lib. Search tab and the Spec List in the Librarian tab are the same list. Spectra may be copied to this list, edited, and deleted from it. Other lists are read-only. All of these lists can be displayed as text row or as graphic structures by selecting the Names or Structures tab at the bottom of the pane.

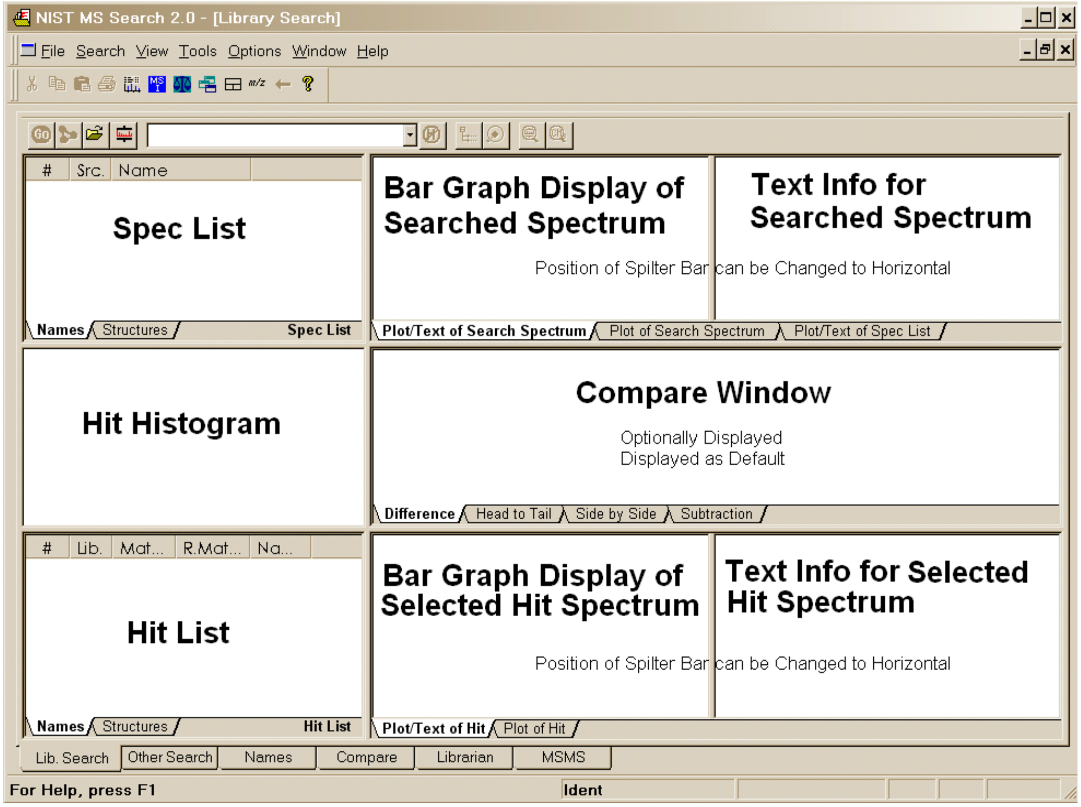

Figure 3. Lib. Search tab.

Placing the Mouse Pointer in one of these panes and clicking the Right Mouse button once will result in the display of a Right Mouse button menu. The last item on this menu is **Properties**. Selecting **Properties** will result in the display of a dialog box (Figure 4.) This dialog box has tabs at the top that are self-explanatory. This allows customization of the appearance of the display and control of what is displayed such as a structure on a spectrum, the 10 most intense peaks in the spectrum in the text display, etc. Colors of structures, mass spectral peaks and axes, and fonts used for alphanumeric characters can also be set. Each pane can have its own customized set of attributes. This customized display can be saved using the **File**/**Save Configuration** command from the **Main Menu Bar**. Different saved configurations can be recalled by using the **File**/**Restore Configuration** command from the **Main Menu Bar**.

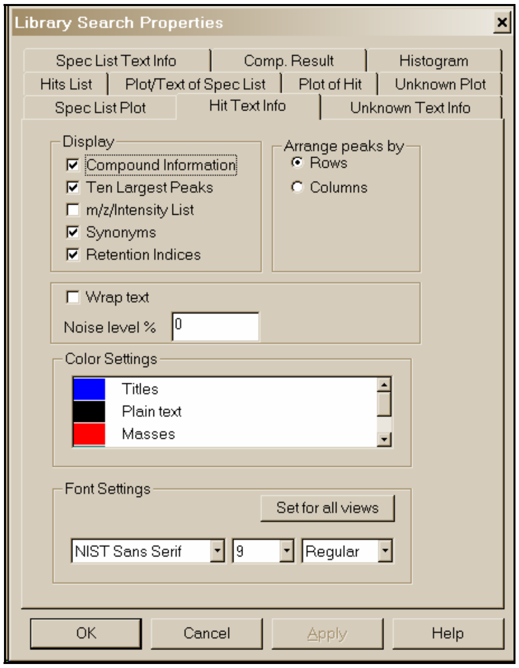

Figure 4. Properties dialog box displayed by selecting Properties on the Right Mouse Button Menu displayed when the mouse pointer is on one of the panes of the LibSearch tab.

The first tab (lower left of the Program's display), **Lib.Search**, is used with a search of a spectrum or structure against mass spectral libraries: a user library or the NIST 08 libraries (Figure 3). The spectrum and structure searches can be done against multiple libraries (maximum of 16), and the selected libraries can be different from those selected for the various searches found in the Other Search tab.

The second tab display, **Other Search** (Figure 5), is used to perform a **Formula, CAS registry number, Molecular weight, Sequential, Any peaks, ID number,** or **NIST number** search. The **NIST number** search can only be done on the NIST main library (**mainlib**) and its replicate library (**replib**). **NIST numbers** establish correspondence between spectra in different releases of the NIST/EPA/NIH MS Library. The **ID number** search can only be done on one library at a time. The other searches in this group can search multiple libraries (maximum of 16), and you can have different sets of libraries for each different type of search.

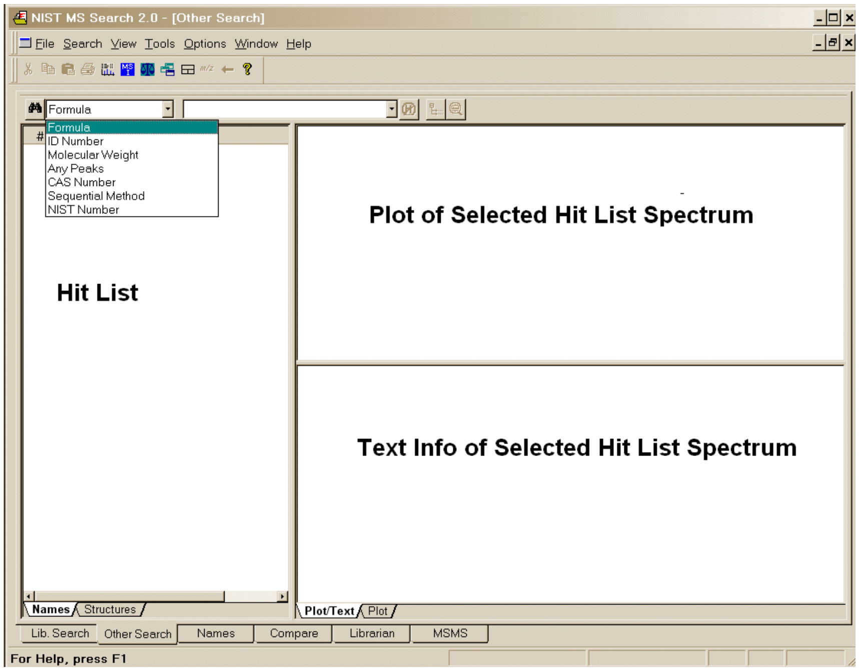

Figure 5. Other Search tab.

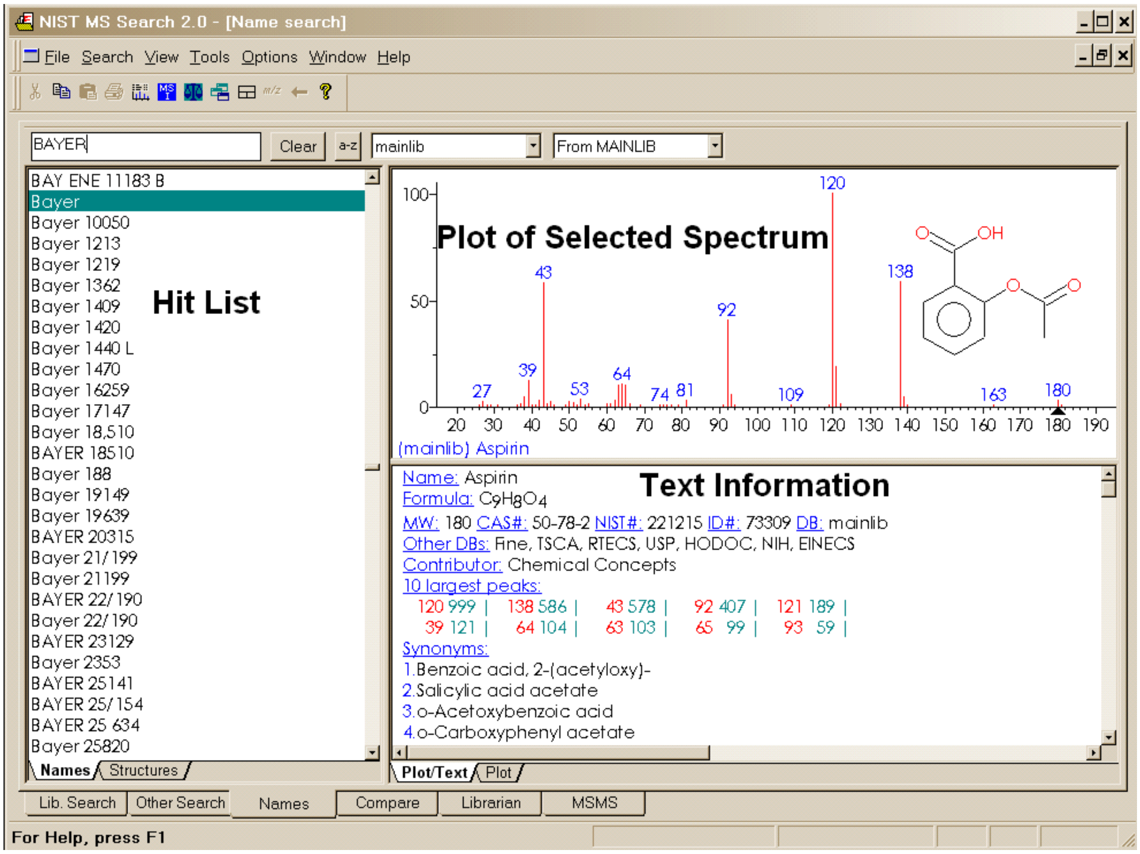

Figure 6. Incremental Name Search tab.

The third tab, **Names**, is used for the NIST signature **Incremental name** search (**INS**) (Figure 6). The **INS** can only be performed against one database at a time. The database to be searched is selected by use of the "library select" drop-down list box next to the **a–z** button. If the **a–z** button is selected, the only input taken is that of letters **a–z**. If the **a–z** button has not been selected, you can type numbers as well as letters. You can also paste Greek symbols. Typed dashes and other punctuation characters will not be used.

The drop-down list box just to the right of the **a–z** button is used to select the library to be searched. This search only allows the use of one library at a time. The second list box is used to display replicate spectra for the **NIST 08 mainlib** library

The fourth tab, **Compare**, allows the display of a sample spectrum and multiple spectra from the Hit List. Other spectra can also be put into the Compare display (Figure 7). A selected spectrum in the lower part of the Compare tab can be displayed as various types of comparisons with the spectrum in the upper part of the display.

The fifth tab, **Librarian** (not shown), is used to deal with spectra that are in user libraries or that are to be added to user libraries. The layout of this tab is the same as the tab used for the Incremental Name Search. The Librarian tab allows for the editing or creating of spectra. This editing not only allows *m/z* intensity pairs to be edited, deleted, or added; but also allows CAS registry numbers, elemental compositions, structures, names, synonyms, and structures to be edited, added, or deleted.

Additional information on the **Librarian** tab is explained later in this manual under **User Libraries**.

When a splitter bar divides a pane, by default, its position is horizontal. This position can be changed to vertical by selecting **Change Splitter Orientation** on the Right Mouse button menu displayed when the pointer is in the pane.

Zoom. Placing mouse pointer on a mass spectrum or spectra compare bar graph, holding left mouse button down and dragging mouse pointer along a diagonal results in drawing a zoom rectangle. After releasing the left mouse button a zoomed spectrum will be displayed; the cursor will change to a magnifying glass. To return to the normal display select **Zoom Out** from the right mouse button menu.

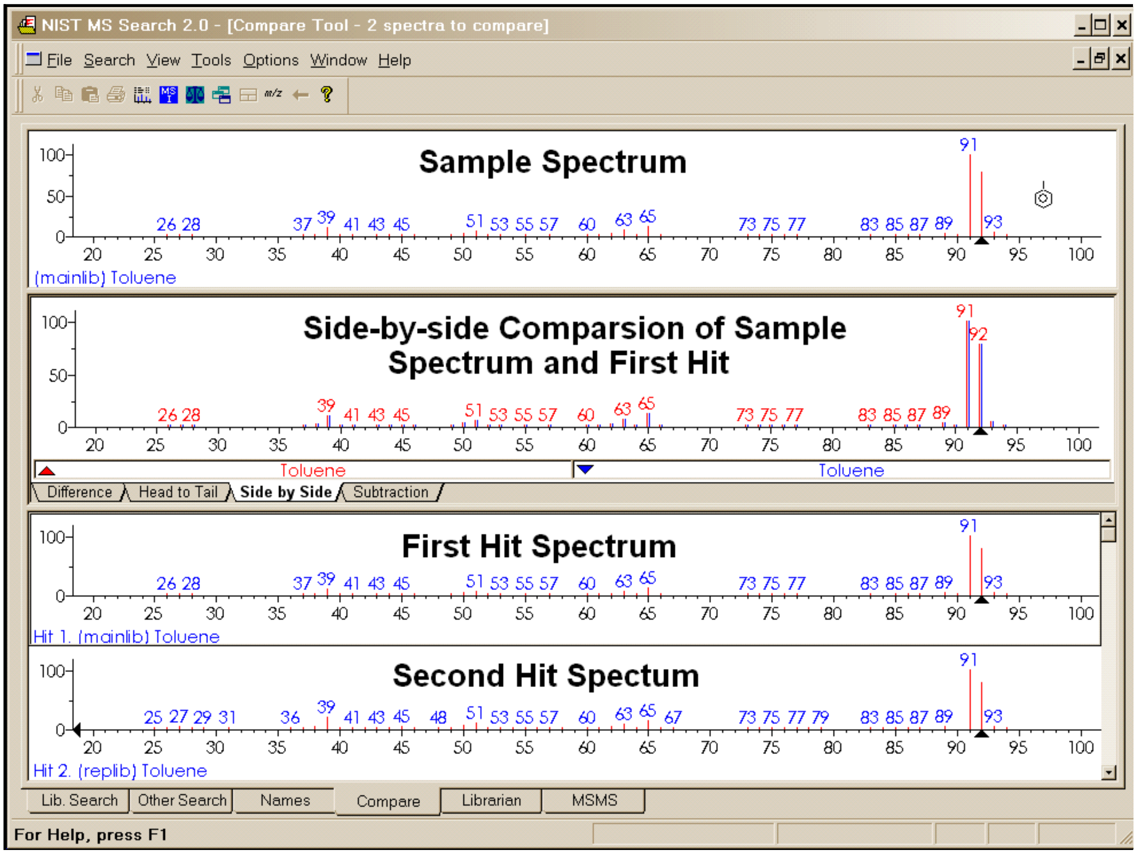

Figure 7. Compare tab.

The Lib. Search Tab

The Lib. Search tab's display (Figure 8) is divided by a vertical splitter bar. All five of the major tab displays have moveable and reorientation divider bars and tabs associated with various windows within a view. In the Lib. Search tab, there are three windows on either side of the splitter bar. On the left side of the splitter bar, the top Window is the **Spec List** (known as the **Clipboard** Window in previous versions of the Program), a **Hit Histogram** in the middle, and a **Hit List** at the bottom. On the right side of the vertical splitter bar are three separate Windows: **Search Spectrum**/**Spec List Spectrum**, top; **Compare**, middle; and **Hit List Spectrum**, bottom. There are vertical splitter bars associated with these six windows that allow for customization of the display. In addition, by selecting **View** from the Main Menu Bar, you can choose to have the **Compare** Window displayed or not displayed. If the Mouse pointer is in the **Search Spectrum**/**Spec List Spectrum** Window or the **Hit List Spectrum** Window, the right mouse button can be clicked to display a menu and select **Change Splitter Orientation** to change the splitter bar between the spectrum plot and text information from vertical to horizontal or back.

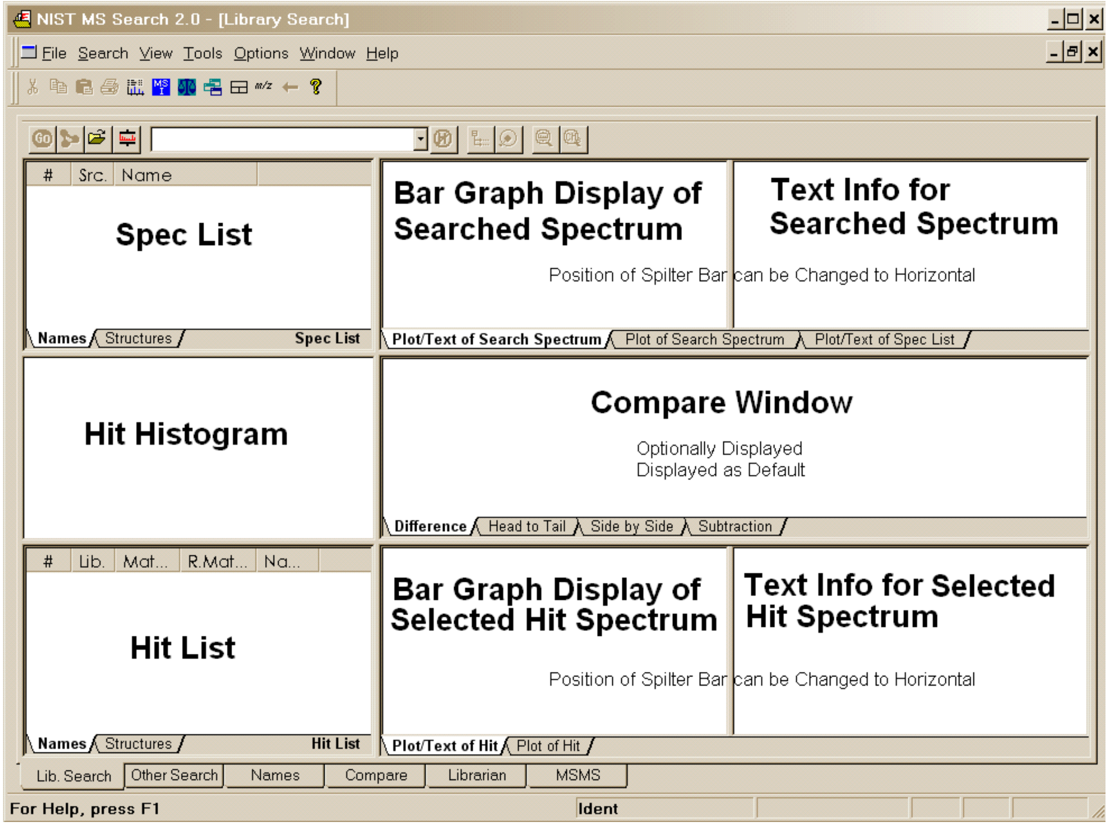

Figure 8. Lib. Search tab.

Just above the Lib. Search tab's window is a button bar (Figure 9).

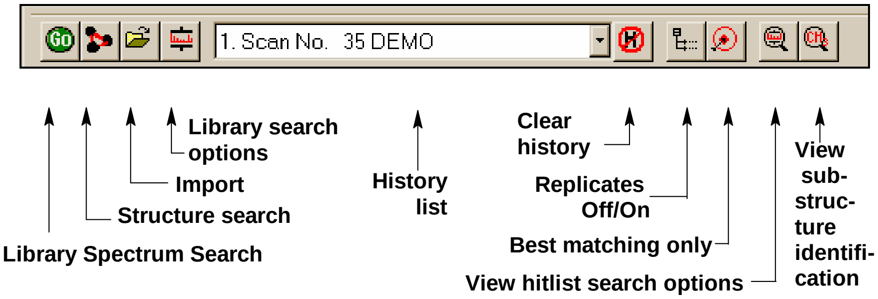

Figure 9. Lib. Search's button bar.

The **Go** button will launch a search of the highlighted spectrum in the **Spec List**. The button to the right of the **Go** button will launch a **Structure** search of the highlighted structure in the **Spec List**. The next button to the right (with the file folder icon) is used to import spectra from a text file. The last button, just before the drop-down list box, is used to display the **Library Search Options** dialog box. This dialog box is used in selecting libraries to be searched, search types, and other factors associated with the search of spectra and structures. The drop-down list box contains the Search History. A previous search's display can be restored by highlighting an entry in this list. **NOTE**: the Program can be set to automatically clear the **Hit List** and/or the **Spec List** by selecting the **Clear History on Exit** *check box* in the Hit List tab and/or Plot/Text of Spec List tab in the **Library Search Properties** dialog box displayed by clicking the right mouse button and selecting **Properties** from the RMB menu. **NOTE**: MS Search v.2.0d used different index files for the **Structure** search in user libraries than previous versions , therefore files created before v.2.0d should be rebuilt using "Rebuild Structure Search Database" from the Tools menu. This should also be done for each new user library connected to the MS Search and every time spectra with structure are added, removed, or edited in a user library because the structure search index files are not updated automatically.

The first button to the right of the “Search History” list box is used to clear the search history (**Clear History** button). The next button (the **Replicates On/Off** button) to the right is used to toggle the listing of replicate spectra on and off when the **mainlib** Library of the NIST/EPA/NIH Library is searched.

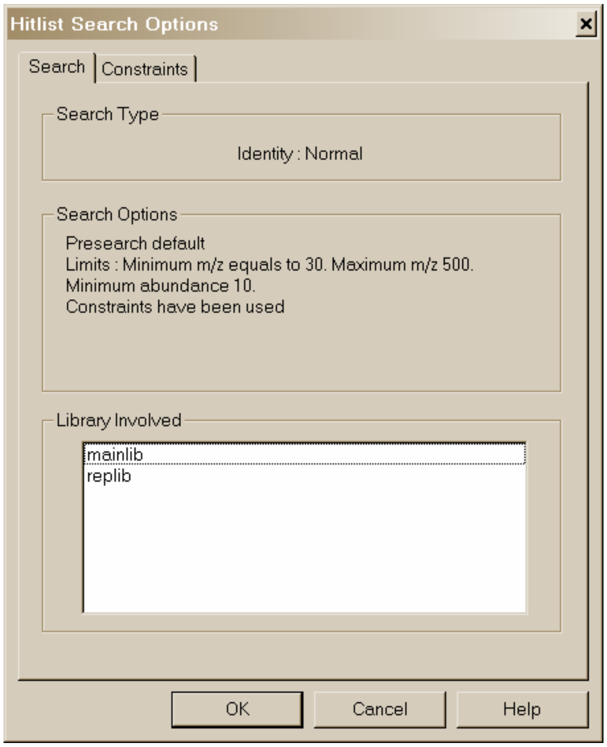

Figure 10. List of options used for the currently displayed search results.

The button to the right of the **Replicates** button is used to display only hits with the highest Match Factor when more than one hit has the same **CAS rn**. This is the **Best matching only** button. This feature is especially useful when more than one library is being searched, and some of the spectra in these libraries have same **CAS rn**’s associated with them.

The button to the right of the **Best matching only** button is the **View hit list search options** button. Selecting **View hit list search options** button will result in the display of a dialog box that lists the parameters of the currently displayed search including library search, constraints, limits, and type of search (Figure 10).

The last button on the right, the **View substructure information** button, displays the **Substructure Information** dialog box. The **Substructure Information** dialog box contains a list of the probability of the presence and absence of substructures that may be associated with a compound that produced the searched mass spectrum along with additional predictions about this compound’s molecular weight and estimate of the numbers or atoms of chlorine and/or bromine that may be contained. This is especially useful with either a Neutral Loss or a Hybrid Neutral Loss “Similarity” search.

Title Bar, Tool Bar, and Status Bar

At the top of the display of each of the NIST MS Search Program’s tab is a **Title Bar**. The contents of a title bar of the active tab is also displayed in the **Title Bar** of the Program window. When the Lib. Search or Other Search tab is displayed, this **Title Bar** will contain information about the currently displayed search. In the case of a User spectrum search, this will include the search type used, whether or not constraints were used, and if any type of limits were applied to the search. In the case of the **Other Search** tab, the **Title Bar** will contain the type of search that was done (i.e., **Formula, Molecular weight, Any peaks**, etc.) and whether or not constraints were used. When the **Compare** tab is displayed, the **Title Bar** contains the number of spectra available for comparison regardless of the number displayed.

At the bottom of Program’s display, below the Program’s tabs, is the optionally displayed **Status Bar**. The **Status Bar**’s display can be turned off by selecting or deselecting **Status Bar** from the **View** menu. When a **User Spectrum** search is being conducted, the left side of the **Status Bar** will have displayed “Comparing XXX Library spectra with submitted spectrum” where XXX is the number of spectra that were found during the presearch (see **Search Algorithms** in Appendix 5). If “Off” has been selected in the Presearch area of the Search tab of the **Library Search Options** dialog box, XXX will represent the total number of spectra in the library(ies) being searched.

There are five fields on the right side of the **Status Bar**. The left-most of these five fields will have displayed the selected search type in the “Spectrum Search Type” area of the Search tab of the **Library Search Options** dialog box (Neutral Loss, Hybrid, or Simple if the “Similarity” search is selected; and Quick or Ident depending on whether a Quick or Normal “Identity” search has been selected).

The next four fields to the right of the box indicating the selected search type pertain to the currently displayed search results. The first box to the right contains the type of search that was used in the displayed search results. The next box to the right will have contained “Rev” if the Hit List is sorted according to the Reverse Match value (the Match Factor obtained by ignoring all peaks that are in the sample spectrum but not the library spectrum). To set this mode select Reverse Search *check box* in the Spectrum Search Options section of the Search tab of the **Library Search Options** dialog box. The third box will contain the number used as a precursor MW value in a

Neutral Loss or Hybrid "Similarity" search. If an "Identity" search has been carried out, the fourth box will contain Penalize if the "Penalize rare compounds" *check box* was selected in the Spectrum Search Options section of the Search tab of the **Library Search Options** dialog box.

The **Tool Bar**'s display (Figure 11) is optional by selecting it from the **View** menu. The functionality of its button **Switch to Caller** and **Print Report** is also available on the **File** menu; the Isotope Calculator, MS Interpreter, and AMDIS also may be launched from the **Tools** menu.

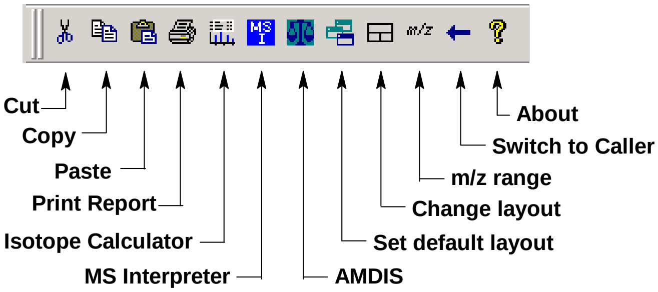

Figure 11. NIST MS Search Toolbar

A Tool Tip is associated with each of the buttons with an explanation of the button's function. The button will return all displays on the currently viewed tab to the default values. The button toggles through three separate views for the currently displayed Program's tab.

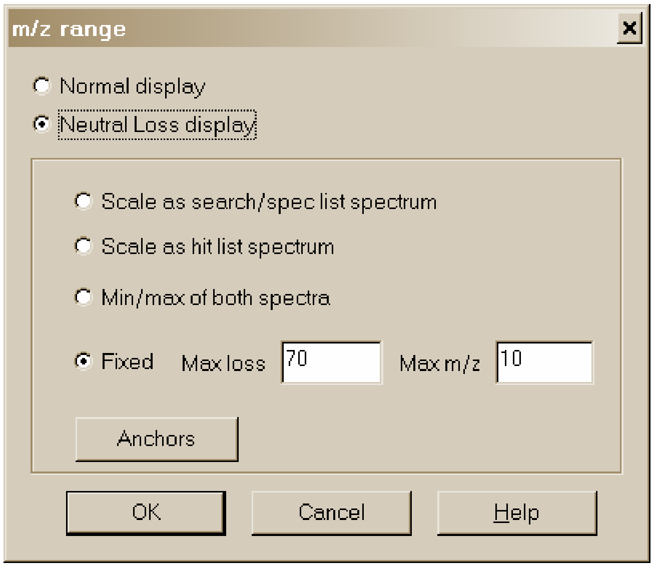

Figure 12. m/z range Spectra Display dialog box.

The ***m/z*** range button displays dialog (Figure 12) to fix the starting and ending *m/z* values for bar-graph spectra displays, and to choose whether the peaks should be labeled with neutral losses or with their *m/z* value.

An explanation of each of the items in this dialog box can be found by selecting the dialog box's **Help** button. The **Anchors** dialog box is used when viewing spectra with a neutral loss label. Selecting **Anchors** will result in the display of the **Anchors** dialog box, which is explained by selecting the **Help** button in that dialog box.

Neutral Loss Display

One of the important tools in deducing a structure from a mass spectrum is to look at the mass spectral peaks as losses from the molecular ion rather than as individual peaks. In this way of viewing the mass spectrum, the peak at *m/z* 269 in methyl stearate becomes the $[M - 29]^+$ peak (loss of an ethyl radical) and the peak at *m/z* 267 becomes the $[M - 31]^+$ peak (loss of a methoxy radical).

A feature of the MS Search v.2.0f is the ability to display a spectrum with neutral loss labeling. If a Neutral Loss or Hybrid search is performed, the sample spectrum and the library spectra may be labeled as neutral losses with the first label on the right-most peak being zero (Figure 13). To activate this display select **Neutral Loss Display** from RMB menu. Selecting Neutral Loss **Anchors** is explained in the Help files (topic m/z range).

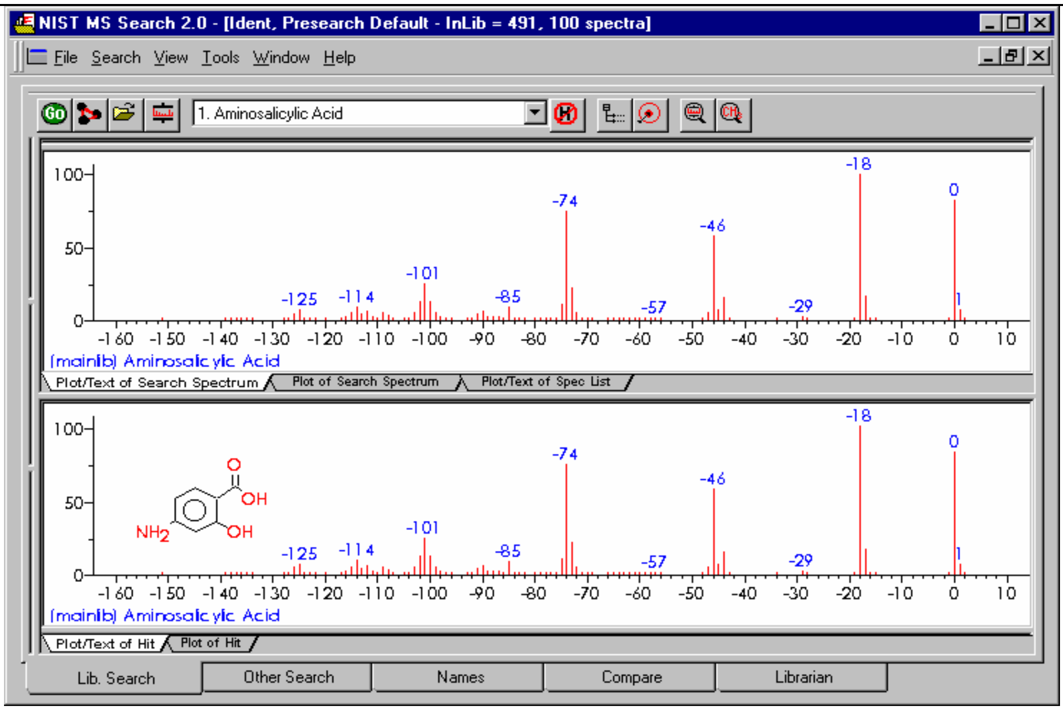

Figure 13. Neutral loss labeling of mass spectral peaks.

On each figure on the top is a sample spectrum; on the bottom is a spectrum of the first library match.

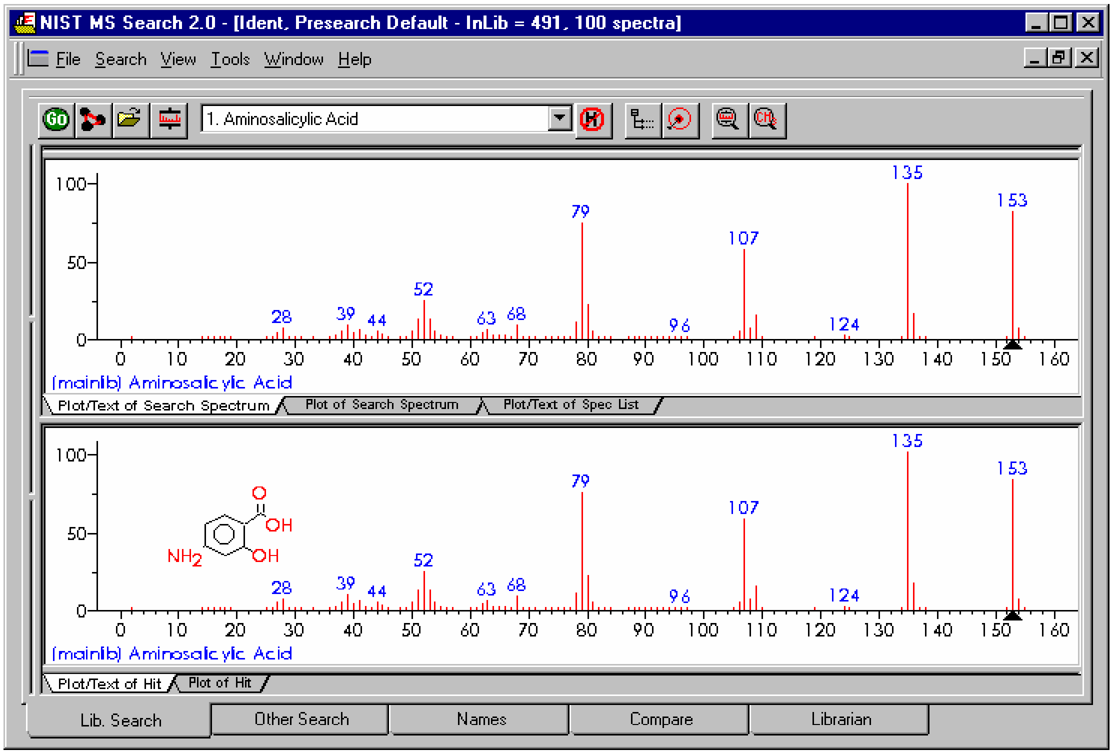

Figure 14. Same display with normal peak labeling.

Spectral Analysis Utilities for NIST 08

Automated Mass Spectrometry Deconvolution and Identification System (AMDIS)

Included as a separate utility, AMDIS attempts to reconstruct original mass spectra for individual components in arbitrarily complex GC/MS and LC/MS chromatograms; and if a target library is provided, ADMIS can directly identify target compounds. AMDIS is especially useful when a single total ion chromatographic (TIC) peak represents multiple components. Regardless of each component's concentration, pure mass spectra are deconvoluted for analyses. AMDIS was developed by NIST under a contract for the Defense Threat Reduction Agency (DTRA), Department of Defense for verifying compliance with a major international treaty (Chemical Weapons Convention) as ratified by the United States Senate in 1997. In order to meet the rigorous requirements for this purpose, AMDIS was tested against more than 30,000 GC/MS data files accumulated by the EPA Contract Laboratory Program without a single false-positive for the target set of known chemical warfare agents. Although this level of reliability may not be required for all laboratories, this shows the degree to which the algorithms have been tested.

AMDIS has been designed to reconstruct "pure component" spectra from complex RTIC chromatograms even when components are present at trace levels. For this purpose, observed chromatographic behavior is used along with a range of noise-reduction methods. AMDIS is distributed with specialized libraries (environmental, flavor and fragrance, and drugs and toxins) that were derived from the NIST 02 MS Library. AMDIS has a range of other features including the ability to search the entire NIST 08 MS Library with any of the spectra extracted from the original data file. It can also employ retention-index windows when identifying target compounds and can make use of internal and external standards maintained in separate libraries. A history list of selected performance standards is also maintained.

The instruments that are supported in their native file format are: [†]

Agilent ChemStation (GC-MS & LC-MS) and MS Engines
BrukerDALTONICS GC-MS
Finnigan (GCQ, INCOS, ITDS, ITS-40, ITD-700/800, ITMS)
Inficon
JEOL /Shrader
Kratos Mach3
Micromass (MassLynx, NT Formats)
Perkin-Elmer TurboMass and ITD-700
Shimadzu MS Files QP5000 and QP5050
Thermo Fisher Xcalibur
Varian (MS, SMS, XMS)
Viking

In addition, the NetCDF format, which is supported by many manufacturers, is supported by AMDIS. Other formats will be added in future releases. Contact NIST for future information.

[†] ***The use of specific product and trade names does not indicate an endorsement by the National Institute of Standards and Technology. The specific file formats are supported in order to ensure that the data is transferred to AMDIS with no information loss. The specific formats and names are the covered by the respective copyrights of the individual companies.***

MS Interpreter

This tool was developed to aid NIST evaluators in their analysis of mass spectra. In one integrated program, it enables a variety of calculations on a mass spectrum, using a proposed chemical structure if available. Spectra and structures are associated in the library-building component of NIST MS Search Program v.2.0 discussed in "User Libraries" section and Appendix 3. MS Interpreter is activated by right-clicking a mass spectrum and then selecting "Send To", "MS Interpreter" or from the **Tools** menu. Peaks in the spectrum originating as a logical fragment of the molecule are marked, and corresponding fragments, derived from thermodynamic approximation, may be highlighted. It also allows the analyst to keep track of important neutral losses, both from the parent or a derived ion and to find all possible formulas for any peak or neutral loss. Isotopic patterns for any cluster can be separately examined. These isotope clusters can be compared to theoretical predictions subject to a number of

user-specified constraints. MS Interpreter is a separate program that also has an icon in the **NIST Mass Spectral Database** program group. It also has a simple Help screen that can be called from the program by selecting **Help** on its Menu Bar or by pressing F1 key. Information about the control currently located under the mouse pointer and possible user actions is displayed in the Status bar located at the bottom of the MS Interpreter main window.

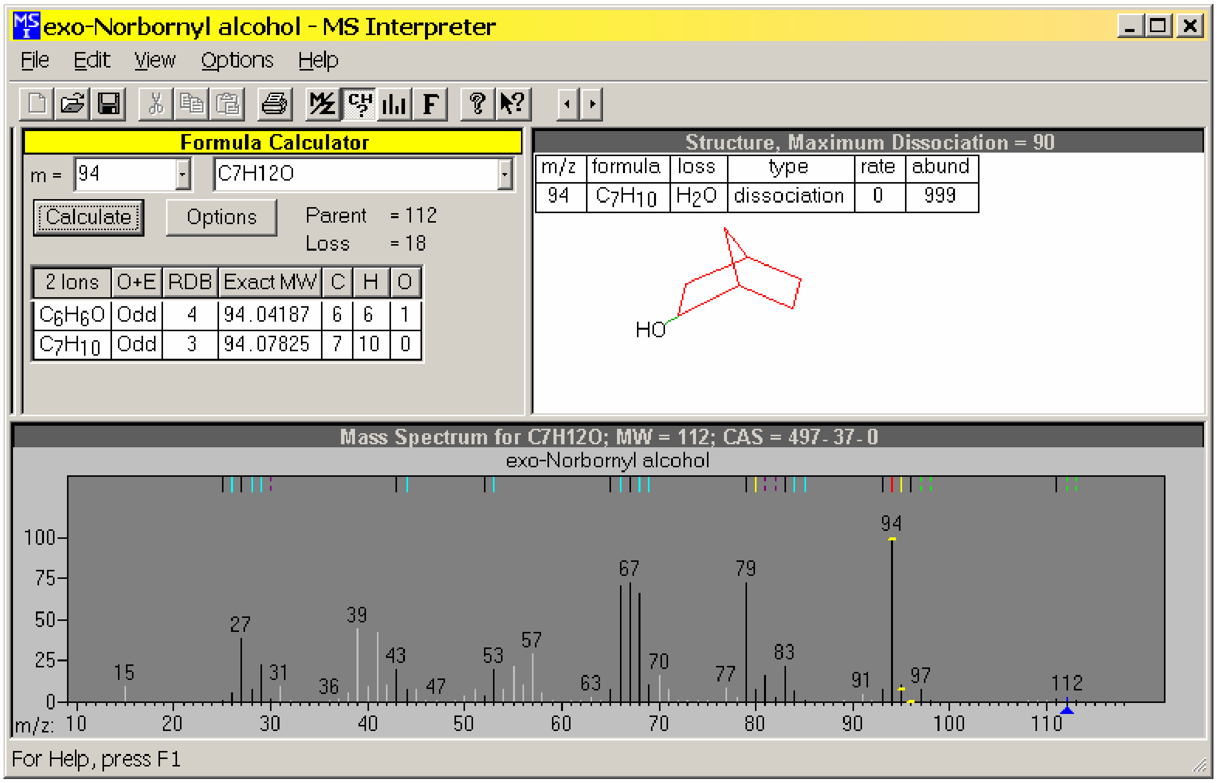

Note, in the above screen, the black lines indicate the peaks in the mass spectrum that the MS Interpreter can explain. The white lines indicate the ones it cannot. The color coding is settable by the user to allow specific types of ion formation mechanisms to be shown.

As shown below, it is also possible to have the MS Interpreter calculate the expected isotope abundance and compare it to the observed spectra.

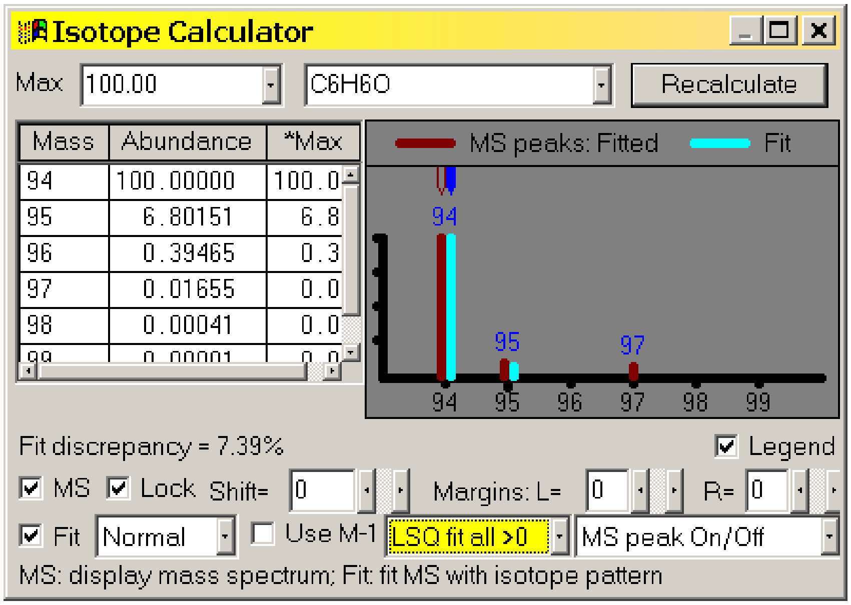

Example Searches

User Spectrum and Structure Searches

This section describes how to use the NIST Mass Spectral Search Program to show how you can find spectra that are similar to that of an unknown compound. This can be accomplished by importing the spectrum from a third-party mass spectral data analysis program or from a text file. You can also use the Librarian to enter the m/z and intensity pairs that make up a mass spectrum. The **User spectrum** search and the **Structure** search are usually carried out from the **Lib. Search** tab display by pressing Library Search or Structure Search button. Another way of starting a search is to select and **click with the right mouse button any spectrum** in a Bar Graph pane or Text Info pane in any tab or any List of spectra or Subtraction spectrum in the Compare Window and select the desirable search from the floating menu. To search for more than one spectrum at once make a multiple selection in any List of spectra (Hit List, Spec List, or Other search list except Name Search List), click the list with the right mouse button, and select Library Search or Structure Similarity Search from the floating menu. Match factor 1000 for the Structure search does not mean an exact match; it means that the found structure is a homologue of the submitted structure. Match factor for the Spectrum Search cannot exceed 999.

Spectra and structures can be imported from files or third-party programs through various types of automations, described later in this section. Spectra or structures can be imported from a file by selecting **File** from the Main Menu Bar and selecting **Open** from the **File** menu (Figure 15). Selecting a file will result in display of the **Structure Import** dialog box or the **Spectrum Import** dialog box. The **Structure Import** dialog box is self-explanatory. The **Spectrum Import** dialog box (Figure 16) is more complex. When a file containing more than one spectrum is selected, one or more selected spectra or all of the spectra can be imported by using either the **Import Selected** or the **Import All** button, respectively. Pressing the **Import Options** button will open the **Spectral Import Options** dialog box (Figure 17). This is where information about the *m/z* values of peaks is controlled.

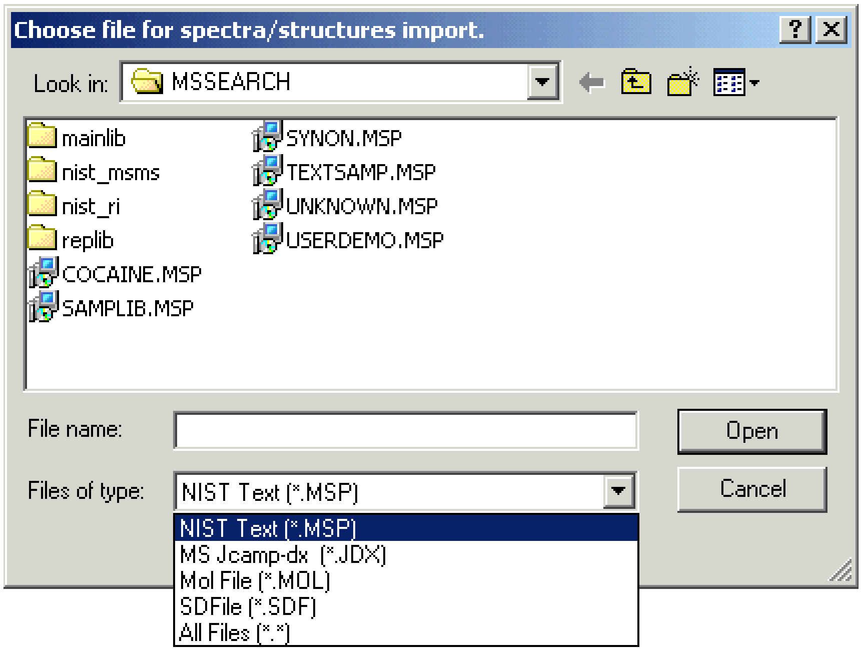

Figure 15. File Selection dialog box with some of the available file types. MSP and JDX files are spectra, and MOL files are structures.

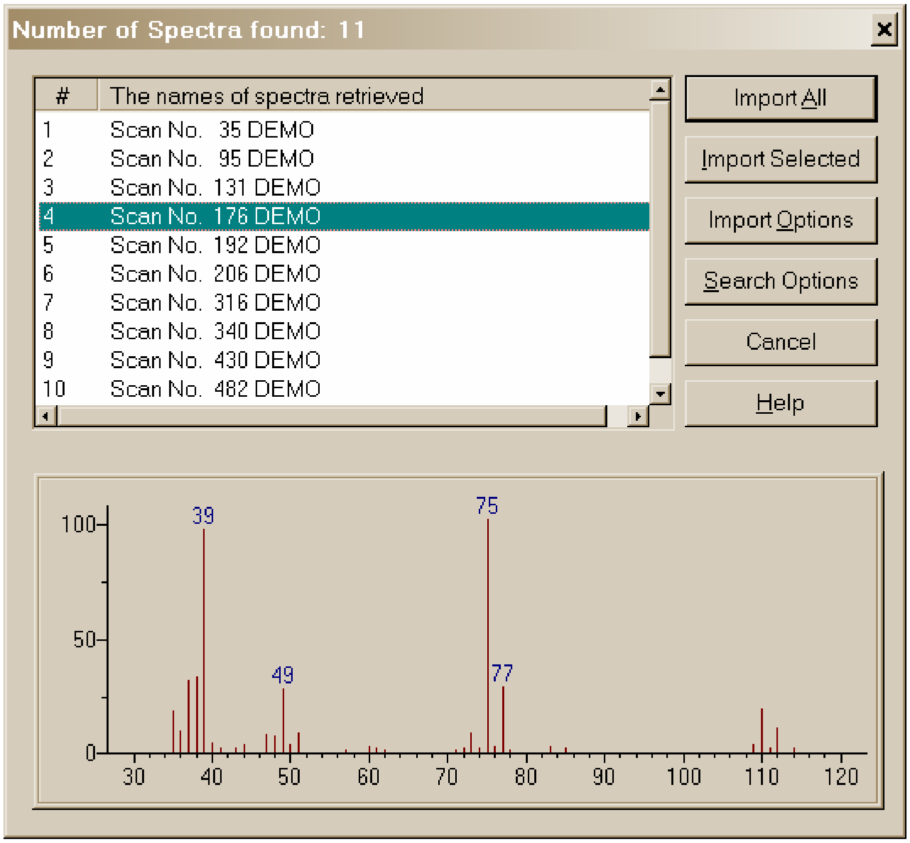

Figure 16. Spectrum Import dialog box with a single spectrum from a multiple spectrum file highlighted and displayed in a bar-graph format.

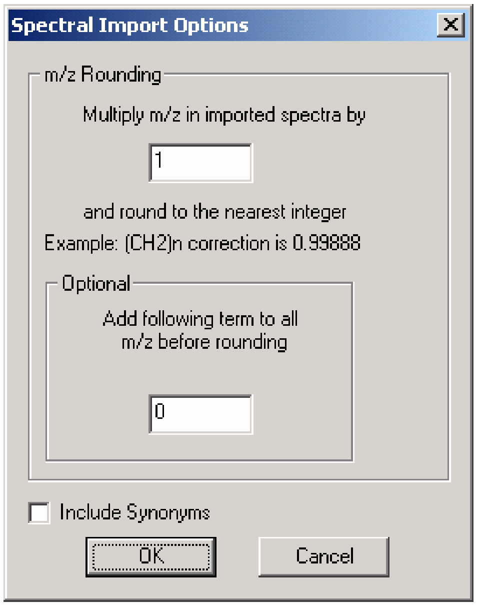

Figure 17. Spectral Import Options Imported spectra integer *m/z* and no mass defect correction.

The two fields in the **Spectral Import Options** dialog box are used to make **mass defect** corrections. The difference in the integer mass and the exact mass of an isotope is the **mass defect**. The most significant effect of mass defect is seen on the integer mass of hydrocarbons due to the **mass defect** of hydrogen. The integer mass of the ^{1}H isotope is 1. The exact mass of this isotope is 1.00725. The $C_{50}H_{100}$ ion has a nominal mass (the sum of integer mass of the most abundant naturally occurring stable isotope of each element times the number of atoms of those elements) of 700, (50 × 12) + (100 × 1). The integer value for $C_{50}H_{100}$ based on a calculation of the exact mass of each of these elements is 701 (12.000000 × 50) + (100 × 1.007825) = 700.7825. The **mass defect** for $C_{50}H_{100}$ is 0.7825, which is equal to about 100 millimass units per 100 mass units. The NIST MS Search value deals with only integer mass values when searching the NIST 08 MS Library. Spectra are imported with integer mass values. Therefore, spectra recorded with observed values of *m/z*, which are not integers and have not been corrected for **mass defect**, must have a **mass defect** correction applied in order to be properly searched against the NIST 08 MS Library or another database that contains only integer *m/z* values.

The mass defect factor can be entered in the box "Multiply *m/z* in imported spectra by" The factor for hydrocarbons based on a general formula of $–(CH_2)_n–$ is 0.99888. When dealing with spectra that have significant mass contribution from multiple atoms of chlorine or bromine (both of which have a significant negative mass defect) a different factor will have to be calculated. In this case, it may be desirous to use the field preceded by "Add following term [value] to all *m/z* values before rounding".

Library Search Options

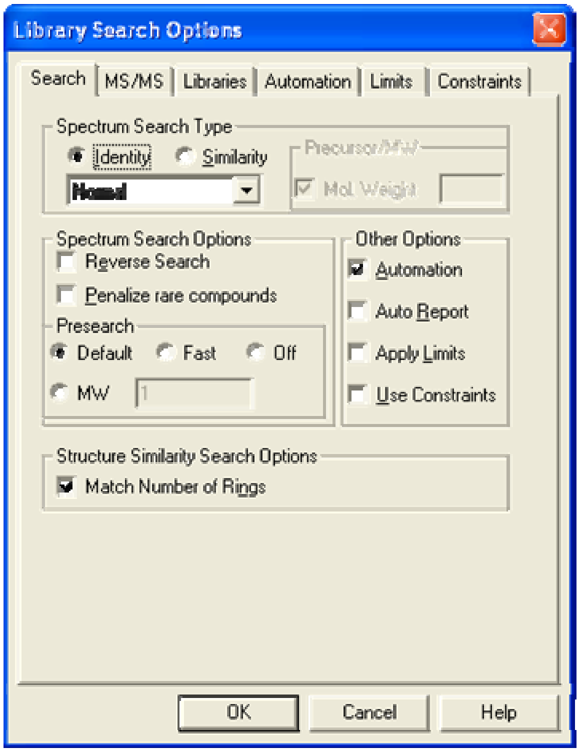

Figure 18. Library Search Options Search tab for an "Identity" MS/MS search

Pressing the Library Search Options button under ***Options*** will display multiple-tab **Library Search Options** dialog box (Figure 18).

A number of different types of spectral comparison routines for choosing the best matching spectra are available. The routine to be used is selected from the **Library Search Options** dialog box's Search tab.

The two search types are "Similarity" (with four options) and "Identity" (with three options). These different searches and their options are explained in the Help Screens (topic Spectrum Search Type).

The Search tab of the **Library Search Options** dialog box also has a **Structure Search constraint** selection. This has the "Match Number of Rings" check box. When selected, the search of compounds that have rings will be constrained to only spectra produced by compounds with a similar structure and the same number of rings as the spectrum being searched.

MS/MS

The MS/MS tab permits selection of the m/z tolerances for the precursor and product ions in the MS/MS Search, the option to ignore the precursor ion, and access to a set of options specific to peptides.

Libraries

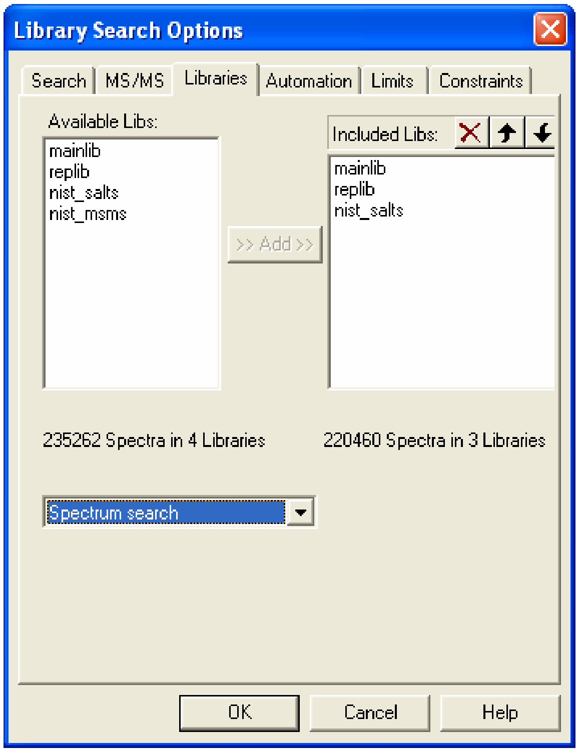

Figure 19. Library Search Options Libraries Tab

You can use the Libraries tab of this dialog box to select the libraries to be included in the User spectrum search. You can have active up to 16 libraries at the same time. This includes the NIST 08 Main Library, **mainlib**, and the NIST 08 Replicates Library, **replib**. By default, three other NIST 08 libraries are also installed, the **nist_salts, nist_ri,** and **nist_msms**. This means that there is only space to accommodate an additional 11 user libraries. If more MS libraries need to be accommodated, the **nist_msms** and **nist_ri** library can be moved to another folder. To add libraries to be searched in the "Available Libs" pane of the dialog box, highlight them and click on the **Add** button. To exclude libraries, highlight them in the "Included Libs" pane and then click on the **X** button above the upper right portion of this pane. The order in which libraries are searched can be changed by highlighting the library to be moved in the "Included Libs" pane and clicking on the Up or Down arrows in the upper right portion of this pane. Each click will move the library one position.

Below the "Available Libs" and "Included Libs" panes is a drop-down list box. This box allows for switching between the lists of libraries to be included in **Structure** searches or **Spectra** searches. These two different types of searches can have different sets of libraries.

Automations

When a **User spectrum** search or a **Structure** search is carried out on a spectrum or a structure that is imported from a file or third-party program such as a drawing program or the instrument data system, a search can be automatically launched. Automatic searching and/or printing can be turned on or off from the Search tab of the **Library Search Options** dialog box by the respective selection or deselecting of the appropriate *check box* (Figure 18).

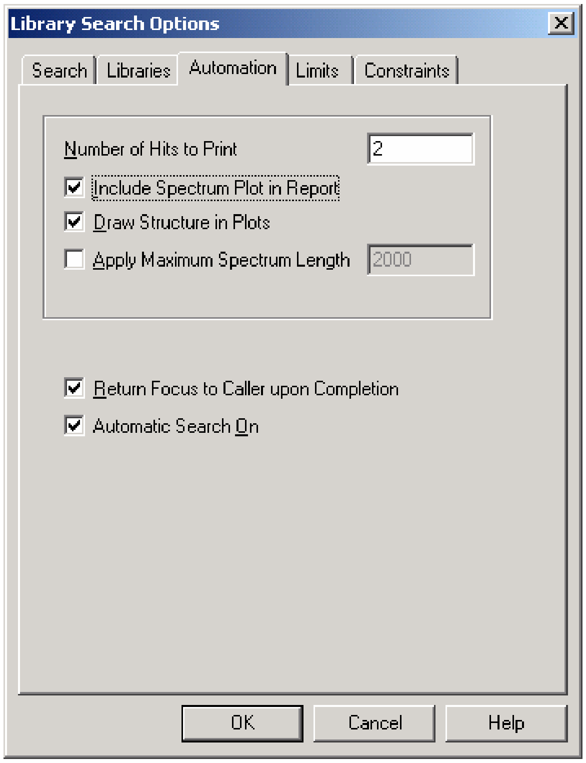

Figure 20. Library Search Options Automation tab.

The **Automation** tab of the **Library Search Options** dialog box is used in conjunction with these automated searches (Figure 20). *Check box* **Automatic Search On** (Figure 20) has exactly same functionality as **Automation** on Figure 18: it turns on automatic searching.

Search results can automatically be printed whether or not the search is automatically launched. *Check box* **Auto Report** (Figure 18) turns on this feature. **Number of Hits to Print** field is located in the **Automation** tab (Figure 20). In addition, the report in the same format can be printed for any displayed search result by selecting **Print Auto Report** from the **File** menu.

The top part of this dialog box pertains to the automated printing of a search result. The format for the Auto Report is a sample spectrum at the top followed by the specified number of Hits in the "Number of Hits to Print" numeric entry box (2 by default). If the "Include Spectrum Plot in Report" *check box* is selected, there will be three spectra per page. Page one will have the sample spectrum at the top followed by the first two hits. Selecting the "Draw Structures in Plots" *check box* will result in the structure associated with a spectrum being printed in the plotted spectrum.

If the "Apply Maximum Spectrum Length" *check box* is selected, a number can be entered for the maximum *m/z* value to appear on the spectrum's abscissa. If peaks are present at higher *m/z* values, the abscissa will have a right-pointing arrowhead at its right end.

Limits

The function of the limits of the starting and ending *m/z* values of a **User spectrum** search and the lowest value of a mass spectral peak that will be considered in a search can be set. An explanation of the limits that can be used to constrain a search may be found in the Help file by selecting the **Help** button on the Limits tab of the **Library Search Options** dialog box.

Constraints

A **User spectrum** search can be constrained by a number of factors such as what (and how many atoms of each) elements must be present in the compounds that produce retrieved spectra, the molecular weight range of hits, whether or not the compounds that produce retrieved spectra are in various other databases, what *m/z* value peaks must be present (and their relative intensity range in retrieved spectra), and what name fragments must be associated with retrieved spectra. These constraints are explained in the Help file, which is displayed by selecting the **Help** button on the Constraints tab of the **Library Search Options** dialog box.

The Search Menu

The Other Search tab's options are:

Option	Description
**Formula**	Finds spectra in selected libraries of compounds with specified formula.
**ID Number**	Finds spectrum (or spectra) in selected library having entered ID number (or range of ID numbers).
**Molecular Weight**	Finds spectra in selected libraries of compounds having specified nominal molecular weight.
**Any Peaks**	Finds spectra in selected libraries based on user-specified peaks.
**CAS Number**	Finds spectra of the compounds that have the entered CAS registry number.
**Sequential Method**	Searches selected libraries using a set of constraints provided by the user.
**NIST Number**	Retrieves a spectrum with the unique NIST Mass Spectral Data Center's Archive identification number (in mainlib and replib only).

These options are also displayed and may be chosen by selecting the drop-down list box on the further most left side of the Other Search tab's display (to the right from the binocular button). Pressing the binocular button starts the currently selected search.

Search by Any Peaks

The **Any peaks** search became very popular when it was first developed over 20 years ago for use with the on-line Mass Spectral Search System from EPA and NIH. This has evolved into the powerful tool that exists in this version of the NIST Mass Spectral Search Program.

Select **Any peaks** from the Other Search tab's menu. This will display the **Any Peaks Search** dialog box. Click the m/z field. The blinking vertical text cursor will be in the "m/z" field. "Normal" will be displayed in "Type" field. *Type 82 and press <Enter>.* This moves the cursor to the "From" field. *Type 70 and press <Enter>.* This moves the cursor to the "To" field. *Type 100 and press <Enter>.* This will enter the peak with its type and intensity range. The cursor is now in the "m/z" field.

Put the mouse pointer on the ↓ on the right side of the "Type" field and click the left Mouse button. Put the Mouse pointer on "Loss" in the displayed menu and click the left Mouse button again. This will cause the cursor to reappear in the "m/z" field. "Loss" will be displayed in the "Type" field. The first peak you are entering is an observed peak in the mass spectrum, and it is possible that it is the base peak. The second peak you are entering represents an ion formed by a loss from the molecular ion. *Type 31 and press <Enter>. Type 5 and press <Enter>. Type 25 and press <Enter>.* The retrieved spectra must have a peak at 31 *m/z* units lower than the molecular weight that exhibits intensity between 5% and 25% of full scale. This reduces the number of spectra in the NIST/EPA/NIH Mass Spectral Main and Replicate Library that match the entered criteria from 1390 for the first peak to 117 that have both peaks.

The next peak will be a "Maxmass" peak. *Click on the ↓ on the right side of the "Type" field. Click on "Maxmass". Type 303 and press <Enter>. Type 1 and press <Enter>. Type 50 and press <Enter>.* There will only be seven spectra that contain all three peaks of the designated type and over the designated intensity range.

Select the ***Search*** *button* in the ***Any Peaks Search*** dialog box to copy the spectra found into the Hit List.

You may also use the Any Peaks criteria in the **Sequential Method Search**. In this case you may also do a search by relative intensity. In this case the subsequent peaks have intensities relative to the first peak. Thus if the first peak has an intensity between 50 and 100% of base, the second peak can be specified to have an intensity of between 40 and 200% of the first peak. This is especially important for anyone using SIM (selected ion monitoring) searches and wanting to understand the rates of false positives.

Search by Name

A useful feature of the NIST Mass Spectral Search Program is its ability to search through hundreds of thousands of names to find mass spectra. When you are given a common name of a compound and want to know if its mass spectrum is in the NIST/EPA/NIH Mass Spectral Library (NIST 08) of approximately 220,000 compounds, you can begin typing the name until it appears in the "Hit List" pane of the Name Search tab. Highlight the desired name, and the display in the **Plot/Text and Plot** Windows will be refreshed with the appropriate displays for the compound.

Use the Mouse pointer to place the blinking vertical text cursor in the "Name" field at the top of the Name Search tab. You are going to search for the mass spectrum of a plant growth regulator, GIBBERELLIN A9. Begin typing the name. Stop after entering each letter. After you have typed the letter N, you will see that the 13th name in the window is your target. You may also paste the name from the Windows Clipboard into the "Name" field by simultaneously pressing keys Shift and Insert.

Sequential Method

In this method every spectrum in the selected libraries is subjected to your search criteria. The criteria that you set in the constraints section govern the hit list returned. In this search method it is possible to use not only absolute but also only relative intensities of peaks. This can be an important tool for examining the risk of false positives when using SIM searches

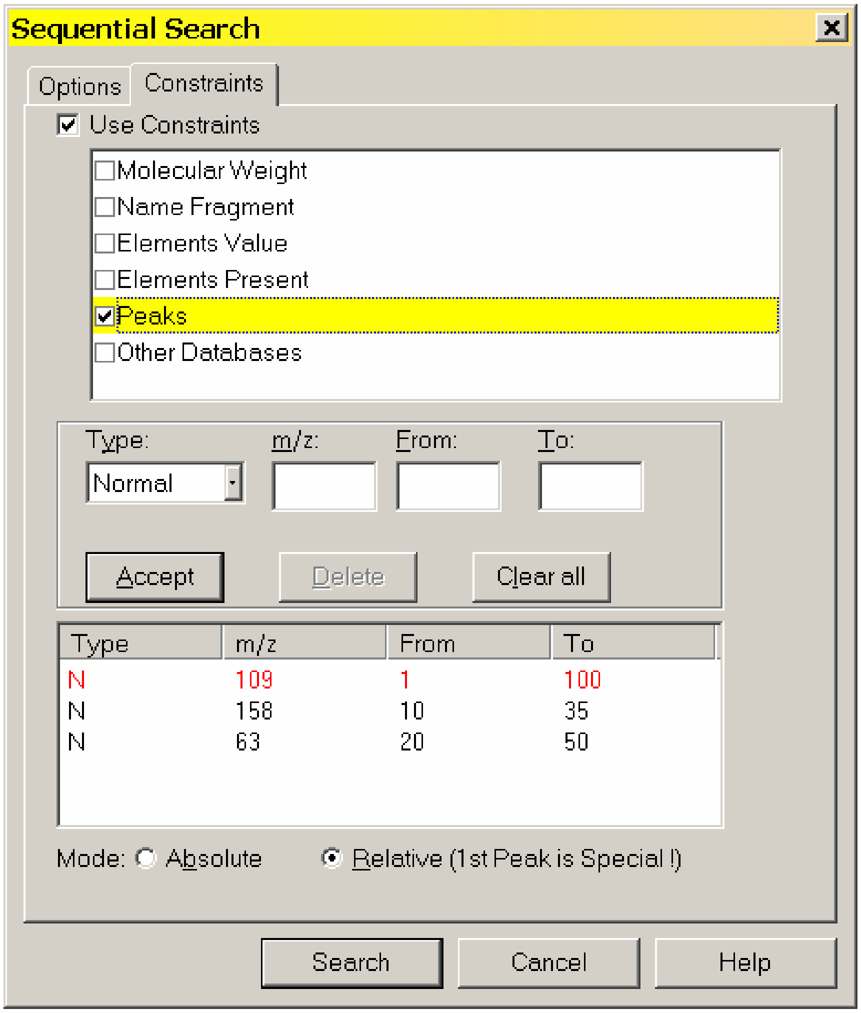

In the example on the left, the peak at 109 is allowed to be present in a range of 1 to 100% of the base peak. The subsequent peaks (158 and 63) are sought as a fraction of the 109 peak, regardless of the intensity of the 109. Thus if the 109 is 15% of base and the 158 is 5% of base (i.e. 33% of the 109) it would be accepted. This kind of analysis can give important insights into to risk of false positive matches when using SIM (selected ion monitoring – also called MIM or multiple ion monitoring) in GC/MS experiments.

In this case the entire spectrum is not seen so the relative intensities of the ions observed are the only data that can be extracted from the data file.

NOTE – all of the other methods of searching are also present in the Sequential Method. The elements search can also be interesting if you want to know (for example) all of the data in the database for compounds that have at least one mercury atom in the molecule. On a modern computer with a large memory these searches do not need to be very slow. Typically any sequential search will be faster for subsequent similar searches since much of the data file will be cached in the computer memory.

Basis for Interpretation of the Library Search Results

When you do an “Identity” search with an unknown spectrum (**User spectrum** search), your results will be associated with three numbers for each spectrum shown and one value for the search. These three numbers are 1) a Match Factor for the unknown and the library spectrum (direct match), 2) a Match Factor for the unknown and the library spectrum ignoring any peaks in the unknown that are not in the library spectrum (reverse search), and, 3) a Probability value (Columns Match, R.Match and Prob., respectively). The first two numbers are fairly straightforward. Each is derived from a modified cosine of the angle between the spectra (normalized dot product). A perfect match results in a value of 999; spectra with no peaks in common result in a value of 0. As a general guide, 900 or greater is an excellent match; 800–900, a good match; 700–800, a fair match. Less than 600 is a very poor match. However, unknown spectra with many peaks will tend to yield lower Match Factors than similar spectra with fewer peaks. The Probability value and the value for the search (InLib, located in the lover right corner of the Hit List and on the Title Bar) require more explanation.

The Probability value for a hit is derived assuming that the compound is represented by a spectrum in the libraries searched. It employs only the differences between adjacent hits in the hit list to get the relative probability that any hit in the hit lists is correct. It was derived from an analysis of the results of searching the NIST/EPA/NIH Main Library with a set of replicate spectra (given in the Replicates Library). Since the total probability of the compound being in the searched libraries is assumed to be one, the relative probability of each of the hits requires only the difference values. The other factor (InLib) is a measure of the probability of the compound actually being in the searched libraries. This was also derived from the same set of replicate spectral searches. In this case, the correct compound was ignored in the hit list; and the difference between the hit lists, with and without the correct compound, was parameterized. The parameters were the maximum value of the match and the largest single difference among the top 20 hits. If the first hit has a high Match Factor (>900) and the next hit has a Match Factor of 800 or less, the probability of the compound being correctly identified is very large and the probability of the compound being in the library is large (the number of hits vs. their Match Factors is displayed in Hit Histogram pane located just above the Hit List, see Figure 8).

Like all statistical results, these probability calculations rely on the data sampled. For example, you may find that a compound that has very few similar compounds (or more importantly very few similar mass spectra) will be identified in a more definitive way. Using examples from the Replicates Library and searching the Main Library using a compound like 'folpet', you will find a high probability for the first hit and a high value for InLib. In contrast, if you take replicates of 'cyclohexanol', you will find that not only are the probabilities much lower but also the InLib value is much lower; and, in some cases, the best match is not even the correct compound. This reflects the fact that there are very few compounds that have mass spectra similar to 'folpet'; but there are a number of compounds that have very similar mass spectra to 'cyclohexanol', and the ability of any search system to distinguish between them is limited. In many cases, the best that the search can do is to provide you with a class of compounds that have similar mass spectra and, usually, similar structure.

The values of the InLib parameter are meant as guideposts. Generally, any positive value is acceptable. Values greater than approximately 300 usually mean that the spectrum is nearly unique. Negative values below 200 are generally a warning that the spectrum is not identified. Note that negative values will occur when there are a large number of compounds with similar spectra. In these cases, the difference between the Match Factors for different spectra is very small, and the search cannot be assured of providing the correct unique answer. Usually in these cases, especially when Match Factors are high, it will provide very good guidance on the structure of the molecule.

For a complete discussion of the methods used in assessing the probabilities, reference Stein, S.E. *J. Am. Soc. Mass Spectrom.* **1994**, *5*, 316–323.

Substructure Information

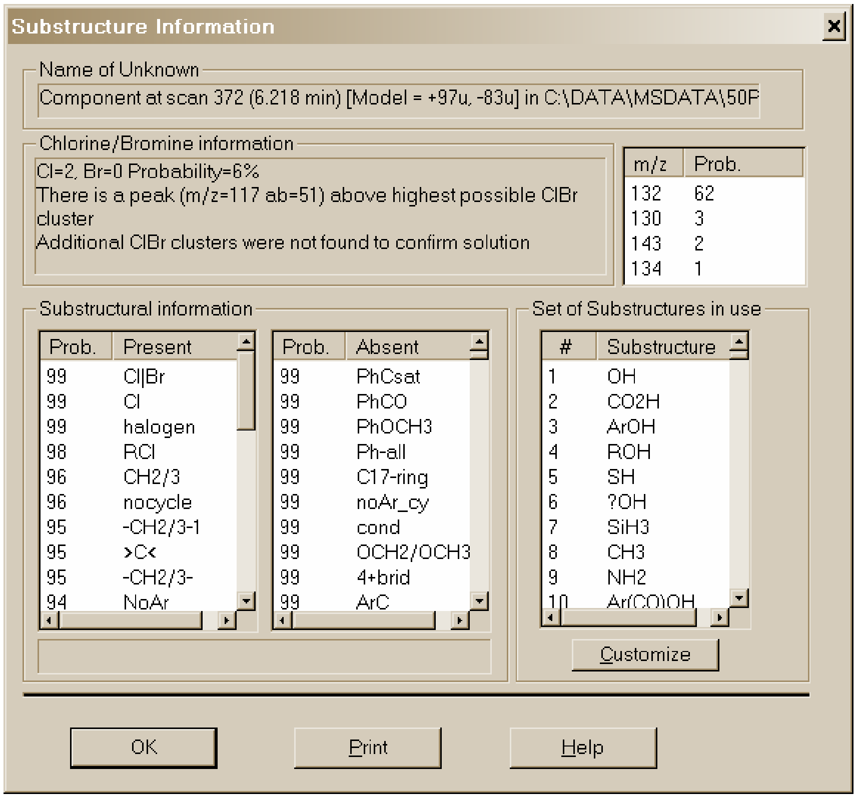

Figure 21. Substructure Information dialog box.

The **Substructure Information** dialog box has the name of the spectrum that was used to create the hit list displayed at the top. There are two panes below this: “Prob. Present” and “Prob. Absent”. These two panes contain a list of abbreviations for the substructures preceded by a number, which is the percent probability for the presence or absence of the substructure. If you do not understand one of the abbreviations, highlight it by using the Mouse and a detailed explanation appears at the bottom of the Substructural information area. In addition, the dialog box contains an area labeled “Chlorine/Bromine Information”, which gives the probabilities and numbers of chlorine and/or bromine atoms present. The result of a molecular weight estimation from the hit list is presented on the right side of the dialog box.

Just below the molecular weight information is the “Set of Substructures in use” pane with a list box and a **Customize** button. The list box displays all the substructures in the current set.

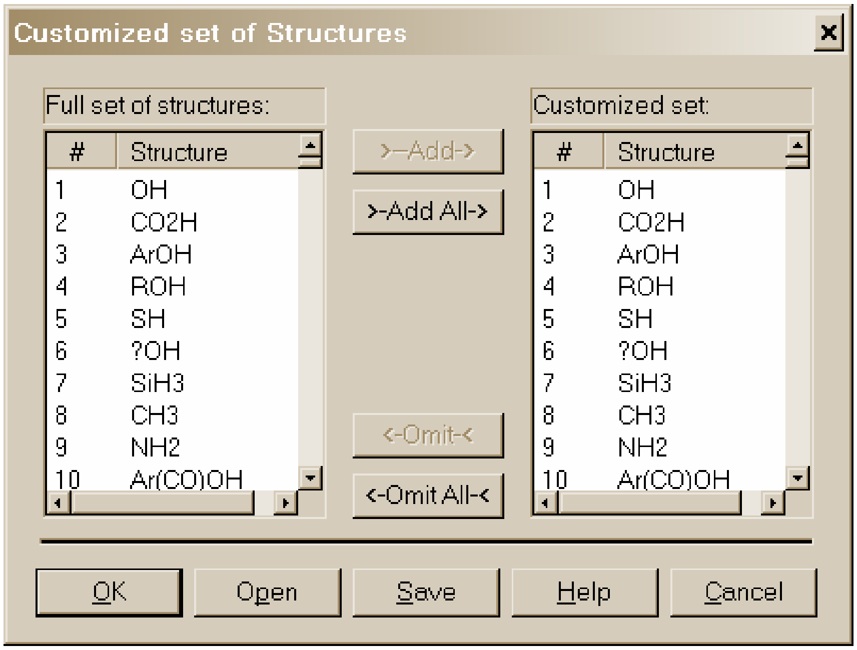

Figure 22. Customized Set of Structures dialog box.

Customize: When this button is selected, the **Customized Set of Structures** dialog box is displayed. This dialog box is used to create a subset of the substructures to be identified. The list of all possible substructures is displayed in the “Full set of structures” pane. The list of substructures included in the present file is shown in the “Customized set” pane. The first time a customized list is created, both panes will contain the same list.

You may omit substructures from the “Customized set” pane or add from the “Full set of structures” pane. Either action occurs on the structures highlighted by Mouse action in the panes by selecting the appropriate button ← **Omit** ← or → **Add** →. The usual Windows conventions for

selecting multiple items apply. After selecting the first substructure of the list, hold down the <Shift> key while selecting the last substructure. This will select all substructures between the first and last. Multiple noncontiguous substructures can be selected by holding down the <Ctrl> key while selecting each desired substructure.

The **Save** and **Open** buttons allow the saving and retrieving of customized substructure sets. A temporary set is created by making modifications to the "Customized set" pane and selecting the **OK** button. This dialog box, as well as the **Substructure Information** dialog box, has a **Help** button, which displays a context-sensitive Help screen.

The algorithms used in the substructure identification are described in the Help screen. They are based on developments at NIST (Stein, S.E. "Chemical Substructure Identification by Mass Spectral Library Searching" *J. Am. Soc. Mass Spectrom.* **1995**, *6*, 644–655).

Use with Instrument Data Systems

The use of the automation features described in the **Automations** section of this manual was implemented to enhance the performance of the NIST MS Search Program when used with proprietary mass spectral data systems.

Location of the NIST MS Search Program

For the implementation it may be necessary to determine the location of the NIST MS Search program files and the location of the NIST MS Search work directory. These may be found in the [NISTMS] section of the WIN.INI file located in the %windir% directory. Below is a typical example of this section:

[NISTMS]
Path32=C:\NIST08\MSSEARCH\
WorkDir32=C:\NIST08\MSSEARCH\
Amdis32Path=C:\NIST08\AMDIS32\
AmdisMSPath=C:\NIST08\AMDIS32\

The string beyond "Path32=" is the path to **MS Search program directory** (location of the executable files); the string beyond "WorkDir32=" is the path to the **MS Search work directory** (location of the history, hits, etc.). Two other items refer to AMDIS and its connection to the MS Search.

Implementation

If your data system is capable of exporting spectra to the NIST format (described in the **USER LIBRARIES** section of this manual) and you can execute a Windows program from within your data system, this feature is available to you. Implementation of this feature has been provided on some data systems.

The command string used to start or bring into focus the NIST MS Search Program for Windows is:

C:\NIST08\MSSEARCH\NISTMS$.EXE <space>/instrument

assuming the program is in the C:\NIST08\MSSEARCH directory; if it is in some other directory, make the appropriate substitution.

Two locator files are required to use the automation features:

1. The first locator file must be in the **MS Search work directory**, have the name AUTOIMP.MSD, and contain the text string describing the name and location of the second locator file. For example:

   C:\MYMSDS\FILESPEC.FIL

   where: C:\MYMSDS is the directory where the file, FILSPEC.FIL, resides.

   This file can be created with any text editor.

2. The second locator file is created each time you save spectra to a separate file to be imported by the MS Search. This second locator file contains the text string that describes the name and location of the text file containing the spectrum (or spectra) in the NIST Text file format described later. You can specify whether the spectra in the NIST MS Search Program's **Spec List** Window are added to or replaced by the automatic import. An example of the contents of the second locator file is:

   C:\MYMSDS\DATA\DATAFILE.TXT<space>OVERWRITE *or* APPEND

   where: C:\MYMSDS\DATA\DATAFILE.TXT is the full pathname of the file containing the spectra, and one of the words OVERWRITE or APPEND is the instruction as to how the spectra are added to the Spec List Window. To enable switching the focus back from the MS Search the second line containing **main window handle numbers** of the data system application may be added (see Figure 23 for details).

   This second locator file is deleted each time the NIST MS Search Program is brought into focus and spectra are imported. Figure 23 illustrates the implementation. The upper part of each box contains a file name, the bottom part is the contents of the file. Black arrows show logical connections between the files.

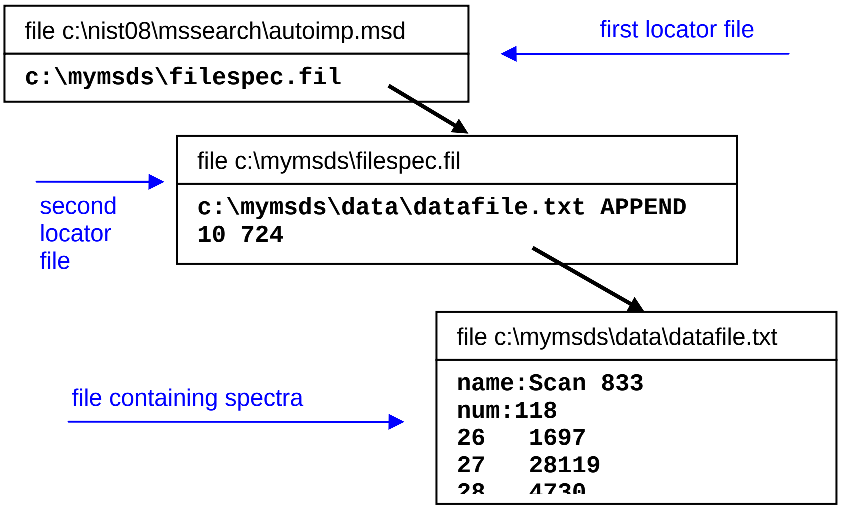

Command line for initiating import of spectra into the MS Search Program:

```
C:\NIST08\MSSEARCH\NISTMS$.EXE /instrument
```

Calculation of the calling program **main window handle numbers**:

hWnd = A02D4(hexadecimal) = HiWord $\times 2^{16}$ + LoWord =

10×2^{16} + 724 (decimal), that is, HiWord = 10, LoWord = 724

(see the 2nd line in c:\mymsds\filespec.fil; the numbers here are for example only; in each run your application may have a different main window handle)

Figure 23. The implementation of use with a data system

**NIST MS Search (nistms$.exe) Command Line Options**	
**Option**	**How it works**
/INSTRUMENT	Normal search. Imports and optionally searches spectra (see Figure 23 for details on the implementation), activates MS Search window, activates switch back button
/INSTRUMENT /PAR=2	Background search. Imports and optionally searches spectra (see Figure 23 for details on the implementation), does not activate MS Search window (in fact, it is disabled during the search), writes hit lists into file SRCRESLT.TXT; when the search is complete creates file SRCREADY.TXT as a signal that SRCRESLT.TXT is ready. Both files are created in the MS Search work directory. Number of hits is determined by the "Number of hits to print" in the Automation tab (Figure 20)
/PAR=4	Start logging. Appends hit lists of Spectrum and Structure searches to the file NISTLOG.TXT located in the MS Search work directory. Number of hits is determined by the "Number of hits to print" in the Automation tab (Figure 20)
/PAR=8	End logging. Stops appending hit lists to the file NISTLOG.TXT.
/MOL="<full pathname>"	Imports chemical structure from a Molfile or SDfile. If SDfile contains more than one Molfile only the first one will be imported. See section "USE WITH THIRD-PARTY DRAWING PROGRAMS" for details.

SEE APPENDIX 1 for instructions on the Creation of AUTOIMP.MSD.

Use with Third-Party Drawing Programs

Version 2 of the NIST MS Search Program provides more support of structures than previous versions. There is a Structure Search Command. If a structure is in the “Spec. List” pane of the Lib. Search tab, this structure can be searched to retrieve spectra of all compounds with similar structures by clicking on the **Structure Search** button (second button from the left) with the structure highlighted in the “Spec. List” pane.

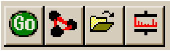

When NIST MS Search v. 2.0 saves a structure to the Windows Clipboard using the right mouse button menu’s command “Copy Structure to Clipboard” the structure is copied to the Windows Clipboard and a file in Molfile format named Clipboard.MOL is written to the NIST MS Search Program’s directory (by default, C:\NIST08\MSSearch). Other programs can either use the structure as it appears on the Windows Clipboard or this file for structure importation.

Structures saved to the Windows Clipboard may be accepted in the MS Search’s Librarian “Spectrum Information” dialog box if its “Clipboard Struct” button is not grayed out. Some drawing programs need to be told how to save a structure in the Windows Clipboard. For example, in ISIS/Draw (MDL Information Systems, Inc.) you need to select “Copy Mol/Rxnfile to the Clipboard” *check box* in Options/Settings/General tab.

If an application is associated with files that have extension .MOL then the structure may be sent to that application by selecting in the right mouse button menu “Send To” and “Default Structure Editor”.

If a spectrum containing a chemical structure is selected, the RMB menu’s command “Send to” will result in a “Send to” menu that can have up to three additional structure editor items. These “Send to” menu items are a function of the first three lines in a text file AUTOIMP.STR located in the NIST MS Search Program’s directory (the WorkDir32 item in [NISTMS] section of the WIN.INI file). Each line should contain three double-quoted strings separated by spaces:

1) A program name as it appears in the “Send to” menu
2) Program executable and path
3) Program startup parameters. %1 will be replaced by the mol file name.

The following is an example of the contents of an AUTOIMP.STR file:

```
"ChemSite" "C:\MolSuite\ChemSite\ChemSite.exe" "%1"
"ISIS Draw" "D:\ISIS Draw\IDraw32.exe" "%1"
"ACD/ChemSketch" "C:\acd800\chemsk.exe" "%1"
```

The last line of the above example results in ACD lab’s ChemSketch starting with the contents of clipboard.mol being imported when ChemSketch opens.

When a program that is to be interactive with the NIST MS Search Program is installed, it must UPDATE or CREATE the AUTOIMP.STR file, or the AUTOIMP.STR file must be edited by the user before the third-party program will appear on and function from the “Send to” menu.

SEE APPENDIX 1 for instructions on the creation of AUTOIMP.STR.

A third-party program can call the NIST MS Search Program by issuing the command:

C:\NIST08\MSSEARCH\NISTMS$.EXE /MOL=<molfile>

assuming the NIST MS Search Program is in the **C:\NIST08\MSSEARCH** directory; if it is in some other directory, make the appropriate substitution (see **Location of the NIST MS Search Program** above)

where <molfile> is a full pathname of the MOL file. If there is a space in the name (e.g., example c:\program files\a.mol), the name should be double quoted:

C:\NIST02\MSSEARCH\NISTMS$.EXE /MOL="c:\program files\a.mol"

A third-party program can obtain the path to NISTMS$.EXE from Path32 in the [NISTMS] section of WIN.INI (see **Location of the NIST MS Search Program** in the “USE WITH INSTRUMENT DATA SYSTEMS” section above).

User Libraries

One of the primary features of the NIST MS Search Program is the ability to do comparisons of user-generated mass spectra and create user libraries to search against. The Program will allow up to sixteen MS libraries, including libraries from NIST 08 MS Library. The user libraries may be searched in conjunction with the NIST/EPA/NIH Mass Spectral Main and/or Replicates Libraries, or they may be searched separately. A maximum of sixteen libraries can be included for search at one time. The Program allows for the searching of combinations of libraries in any order. The inclusion of libraries and their order of search can be set from the **Libraries** tab of the **Library Search Options** dialog box displayed (Figure 19).

User libraries are maintained as subdirectories under the MS Search Program's root (work) directory. The name given to the user library is the name of the subdirectory. This subdirectory will contain a number of database files and indexes constituting the user library. These files exist in a proprietary format. The spectra and compound information are put in these files through the **Librarian** tab or by using Lib2NIST library utility.

The spectra and compound information can originate in a text file in the NIST Text file format described in the **NIST Text Format of Individual Spectra** section of this manual. Spectra can also be entered through the Program. This is done by using the **Spectrum Information** dialog box called with the **New spectrum** button in the **Librarian** tab toolbar.

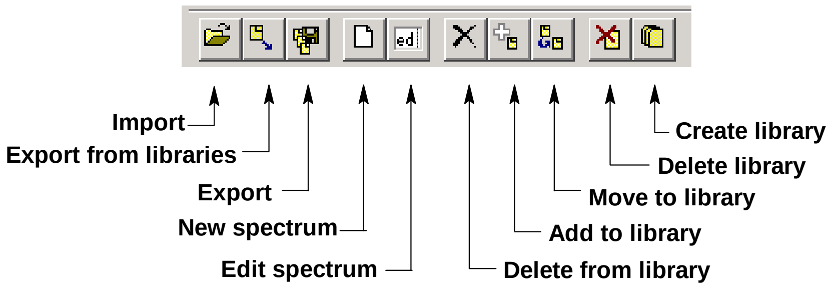

Figure 24. Librarian Toolbar

User libraries are easy to create and maintain. You may want to create them using specific sets of spectra from the NIST/EPA/NIH Main or Replicates Library. Any spectrum in the **SpecList** can be added to a user library. Any spectrum in the **Hit List** Window, Other Search list, Names list, Bar Graph window, Text Info window, or Subtraction pane in the Compare window can be copied to the **Spec List** Window by selecting "Send To", "Spec List" from the right mouse button menu. The size of a user library is limited by 65,535 spectra.

The MS Search Program can read user-submitted spectra in the JCAMP, U.S. EPA, or NIST Text file formats as well as formats of files submitted by various instrument manufacturers' mass spectrometry data systems. You should check with the manufacturer of your mass spectrometer's data system to see if there is file compatibility.

Instructions on building user libraries are included in the **Librarian** Help screens. These also include the information necessary to add structures to user library spectra.

NIST Text Format of Individual Spectra

The NIST Text file format is simple. A file can contain as many spectra as you want. Each spectrum must start with the field title “Name:”. There must be something in this field in order for the spectrum to be imported (i.e., the datafile name and the scan number of the spectrum’s fetus). The next required field title is “Num Peaks:”. The contents of this field must be the number of mass/intensity pairs that make up the spectrum. Optional fields with the titles of “Comments:”, “Formula:”, “MW:”, “Synonym:”, and “CAS#:” can be between the “Name:” and “Num Peaks:” fields.

When a spectrum is saved to a text file from the NIST/EPA/NIH Main or Replicates Library, it will also contain fields with the names “NIST#:” and “DB#:”. The field with the name “NIST#:” is on the same line as the “CAS#:” field and separated by a semicolon (;). “DB#” is same as the ID# displayed in the spectrum text window. The mass/intensity list begins on the line following the line with the field title “Num Peaks:”. The lines of the mass/intensity list have no field title.

When you create a file with all the allowable fields, each field title should be on a separate line. Use the following format. The format for each spectrum in a text file usable by the Program should be as follows:

Line 1: **NAME: Molecule** (Required, up to 511 characters)
Line 2: **COMMENT: Run 23, 8/8/88** (Optional, up to 1023 characters)
Line 3: **FORMULA: C6H6** (Optional, up to 23 characters)
Line 4: **MW: 78** (Optional)

If the **User spectrum** search contains a CAS registry number with its appropriate prefix (**CAS:**), the display of the spectrum in the **Plot**, **Compare**, and **Structure** Windows will have the structure of the compound with that CAS registry number if it is present in the NIST/EPA/NIH Main Library.

Line 5: **CAS: 71-43-2** (Optional)
Line 6: **SYNONYM: Chemical name synonym** (Optional, may be repeated)

The actual mass spectral data (number of peaks in the spectrum) must begin on the next line. It does not actually matter what line this is, as long as it precedes the line that starts the mass/intensity pair data. The VERY NEXT line and subsequent lines MUST contain the paired mass/intensity values.

Line 7: **Num Peaks: 18** (This prefix and the exact number of mass/intensity pairs is Required.)
Line 8: **26 430; 27 340; 28 40; 37 480; 38 611; 39 1411; 49 300; 50 1792;**
Line 9: **51 2052; 52 1962; 63 340; 73 160; 74 480; 75 180;**

…

Line n: **76 721; 77 1401; 78 9806; 79 651;**

The peaks need not be normalized, and the masses need not be ordered. The exact spacing and delimiters used for the mass/intensity pairs are unimportant. For example, the peaks above could equally be presented as:

(26,430),(27,340),(28,40),(37,480),(38,611),(39,1411),
(49,300), (75,180),(50,1792),(51,2052),(52,1962),(63,340),
(73,160),(74,480),(75,180),(76,721),(77,1401),(78,9806),(79,651)

or each mass intensity pair could be on an individual line. The following characters are accepted as delimiters (except “|”)**: |space|tab|,|;|:|(|)|[|]|}|**

You can give the file any valid file name. However, it is best to use the extension “MSP”. This is the recognized default extension in the MS Search Program when you want to import user library spectra.

Several examples of NIST text format mass spectral files are installed together with the MS Search Program, namely SAMPLIB.MSP, UNKNOWN.MSP, and USERDEMO.MSP.

APPENDIX 1: Creating AUTOIMP Files Used in NIST MS Search V.2.0

It may be necessary for you to create AUTOIMP.MSD as the primary locator file used for the importation of spectra from third-party mass spectral data systems and/or AUTOIMP.STR for interaction between the MS Search Program and third-party drawing programs. If you are using Microsoft Windows 2000, Windows NT 4.0, or Windows XP you may create these files using Notepad.

If you use Microsoft Notepad, the default extension is .TXT (Text Document) even though you type AUTOIMP.MSD or AUTOIMP.STR. The default settings for Windows Explorer will not show this .TXT extension. The file will not work unless the .TXT extension is removed. You can rename the file by highlighting the file in Windows Explorer and selecting “Rename” from the **File** menu.

When saving the file using Save As option this problem may be completely avoided by selecting “**All Files**” in the “Save as type” drop down list box. In this case, the file will be saved with the extension provided by you in the “File Name” field (see the snapshot of the Save As dialog box below).

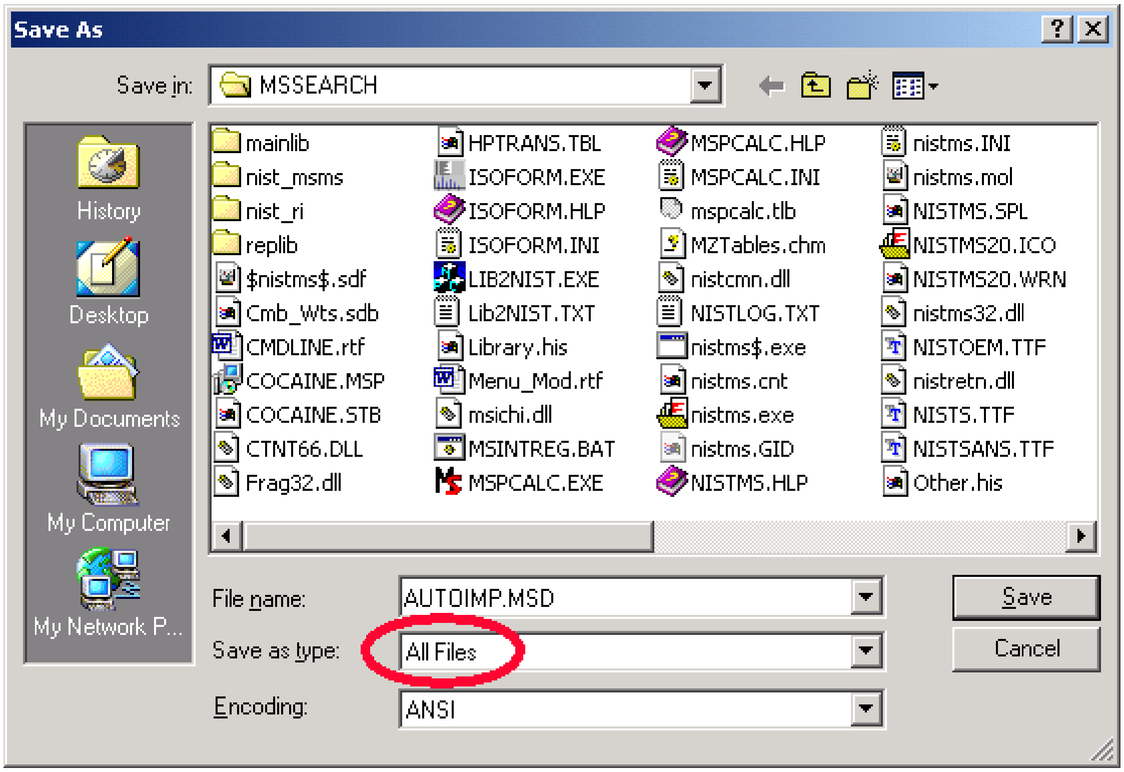

When saving the file using the Notepad “Save As” option, also make sure the **ANSI** Encoding is selected (in some versions of Windows the Encoding option is not present).

After File name, Save as type, and Encoding are set correctly, press the Save button.

APPENDIX 2: Using the NIST MS Search Program with ChemStation

The NIST MS Search Program has been supported through several versions of the HP/Agilent Technologies ChemStation. In the recent versions of the ChemStation for GC/MS, both the **NIST Output** and **AMDIS** options will appear on the **Spectrum** menu of the Data Analysis software when using the Enhanced Quantitation, Standard Quantitation, or Gasoline mode; and on the **Lib** menu when using the Environmental mode if the appropriate macros have been installed. The NIST/EPA/NIH MS Library (NIST 08) setup will install the two required macros along with additional macros that will allow for greater flexibility with the ChemStation software.

It is possible to use the ChemStation double-click option on a displayed spectrum to send that spectrum to the NIST MS Search Program's **Spec List** Window, bringing the MS Search Program into focus. If the appropriate options (including Automation, see Figure 18 and Figure 20) have been set in the NIST MS Search Program then, after the spectrum is automatically prepended to the **Spec List** Window, it will be searched.

If you have an Enhanced Quantitation mode on your version of ChemStation, the setup will automatically add four new options to the **Spectrum** menu (**PBM Quick** search, **NIST** search, **Multiple Lib PBM** search, and **Select Default Search Engine**). Each of these menu options is explained below. If you want to add these options to other modes of the ChemStation software see the **MENU_MOD** document file on the NIST MS Search Program installation CD ROM in the \support.cs directory. This document is also available after installing NIST 08 MS Library by selecting **ChemStation Menu Modification** from the **NIST Mass Spectral Database** program group.

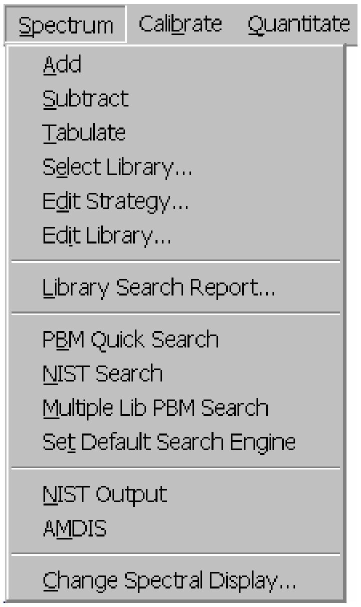

Some older versions of ChemStation will have **ASCII Output** as opposed to **NIST Output**, the **Spectrum** menu, or the **Lib** menu found on the Environmental mode. Selecting either the **NIST Output** or **ASCII Output** results in the display of the **Options for NIST Library Search** dialog box with six *radio button* options and **OK** and **Cancel** buttons. The Macro NISTINT.MAC must be installed in the HPCHEM\MSEXE or MSDCHEM\MSEXE directory for these menu selections to be displayed.

When all the necessary macros have been installed in the ChemStation's MSEXE directory and the modifications have been made to the ChemStation macros, the following options will be available:

The **PBM Quick** search (PBMSrch.mac) performs the normal ChemStation PBM search of the currently displayed spectrum.

The **NIST** search (NISTSrch.mac) passes the currently displayed mass spectrum to the NIST MS Search Program. The spectrum will be prepended to the NIST MS Search Program's **Spec List** Window. If the NIST MS Search Program is configured to search automatically, then the search will be started when the spectrum is passed to the NIST MS Search Program. Otherwise, you can double click on any spectrum in the **Spec List** Window located in the Lib. Search tab to start a search.

The **Multiple Lib PBM** search (LS.mac) performs a ChemStation PBM search using up to three different libraries (all of which have to be specified using the dialog box displayed by selecting the **Select Library** option on the **Search** menu). The top 20 hits from each library will be displayed in order of decreasing match quality.

Set Default Search Engine (SWITCH.MAC) displays a dialog box that allows you to select the default library search to be evoked when you double click the RMB on the currently displayed spectrum. The standard PBM search is default until you select another default.

NIST Output (NISTINT.mac) displays a self-explanatory dialog box that allows for the use of the NIST MS Search Program in a number of different ways and the copying of ChemStation user libraries as NIST MS Search Program user libraries.

AMDIS (AMDIS.mac) loads AMDIS with the data file currently being viewed with the ChemStation Data Analysis directly into AMDIS for analysis. Once in AMDIS, select **Run** to perform the analysis.

The **Lib2NIST** converter program will be installed along with the NIST MS Search Program. This program allows you to copy mass spectral libraries that have been created using the internal utilities of the ChemStation software (including structures) and other mass spectral libraries to user libraries that can be employed by the NIST MS Search Program. This utility is evoked as an option from the **Options for NIST Library Search** dialog box

displayed by selecting the **Copy ChemStation Lib to NIST Format** *radio button* and then selecting the **OK** button or by selecting the **Lib2NIST** icon in the **NIST Mass Spectral Database** program group.

An additional macro is found on the NIST MS Search Program's installation disk, **AUTONIST.MAC**. This macro is to be incorporated into automated sequences. It automatically selects spectra based on the autointegration of the current data file and switches to the NIST MS Search Program where the spectra are automatically imported into the **Spec List** Window. If the proper settings have been made in the Program, these spectra are searched and the focus returned to the HP MS software. You must be using G1034C, Version C.3.00.00 or later of ChemStation to utilize these features.

Additional Information Regarding Modes Other Than Enhanced Quantitation

During the installation of the NIST/EPA/NIH MS Library (NIST 08), if you have selected the NIST MS Search Program as your ChemStation default search, each time you double click on a displayed spectrum, the spectrum will be prepended in the NIST MS Search Program's **Spec List** Window. The NIST MS Search Program will be brought into focus *regardless* of the data analysis mode (Enhanced Quantitation, Standard Quantitation, Gasoline, or Environmental). You can change to one of the other above-referenced search methods by selecting the **ChemStation Default Search Mode** icon in the **NIST Mass Spectral Database** program group.

YOU DO NOT HAVE TO MODIFY ANY MACROS TO SWITCH FROM NIST MS SEARCH PROGRAM AS THE DEFAULT SEARCH METHOD FOR THE DOUBLE-CLICK FEATURE OF THE ChemStation.

HP_NIST MACRO (NISTINT.MAC)

When you select **NIST Output** from the **Spectrum** menu of the MS ChemStation or **ASCII Output** from the **Lib** menu of the Environmental software, the **Options For NIST Library Search** dialog box is displayed. This dialog box consists of six options and **OK** and **Cancel** buttons. Each option has a *radio button* next to it. Use the Mouse pointer and the Left Mouse button (LMB) on the *radio button* to select an option. Selecting the **OK** button results in execution of the option.

Items in the **Options For NIST Library Search** dialog box are selected by putting the Mouse pointer on the appropriate *radio button* and clicking the LMB. Then select the **OK** button to execute the desired option. If any of the first three options is executed, the dialog box will be redisplayed.

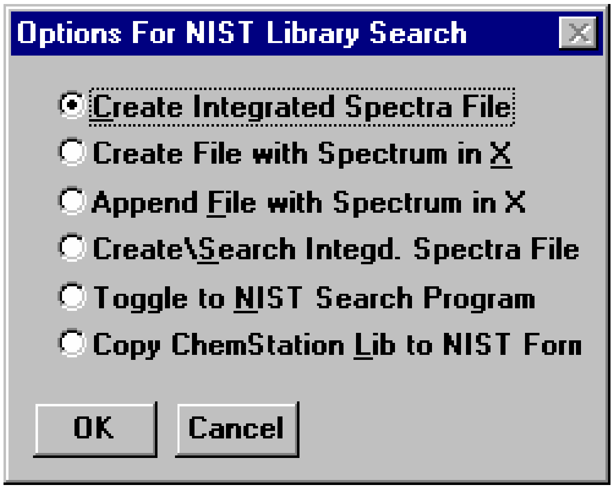

Result of s*electing* **NIST Output** from the **Spectrum** menu

Each time one of the top four menu selections is executed, the file HPNIST.TXT is created in the ChemStation HPCHEM or MSCHEM directory. This file contains the name and location of the text file that contains the saved spectra in the NIST format. The HPNIST.TXT file is deleted every time the NIST MS Search Program is brought into focus and spectra are automatically imported into the top of the NIST MS Search Program's **Spec List** Window.

At the same time the HPNIST.TXT, the second locator file, is created, a text file named TEMPSPEC.MSP containing one or more spectra is created or appended. The text file is located in the ChemStation directory HPCHEM or MSDCHEM. This text file will remain there until it is overwritten or removed manually.

When the NIST MS Search Program comes into focus, the spectra that have been saved in the ChemStation software are automatically copied to the **Spec List** Window. If spectra are already in the **Spec List** Window, you are prompted to **Prepend** or **Overwrite** the current contents. If you select **Prepend**, the new spectra are added to the top of the **Spec List** Window (prepended). If you select **Cancel**, the automatic import will be aborted. However, the HPNIST.TXT file may be deleted even in the case of an abort. If you have selected the options of **Automatic Search On** and **Return Focus to Caller upon Completion** in the **Automation** dialog box, selecting **Cancel** returns the focus to the ChemStation software.

Menu “Options for NIST Library Search”

The **Create Integrated Spectra File** option runs the ChemStation’s Autointegrate routine on the displayed chromatogram and creates the text file containing the spectra that represent the apex of each chromatographic peak.

The **Create File with Spectrum in X** option creates a text file that contains the spectrum that is in X of the ChemStation Stack display. This is where you will find background subtracted and averaged spectra. This is the currently displayed spectrum.

The **Append File with Spectrum in X** option appends the spectrum in X of the Stack display to the existing text file. If a text file does not exist, one will be created.

The **Create\Search Integd. Spect. File** option creates a text file that contains spectra representing the tops of each chromatographic peak and either starts or switches to the NIST MS Search Program where the spectra are automatically imported into the **Spec List** Window. If **Automatic Search On** in the NIST MS Search Program’s **Automation** dialog box has been selected, each spectrum will be searched. The results are optionally printed, and the results of each search are stored in the Hit List History. If **Return Focus to Caller upon Completion** has been selected in the **Automation** dialog box then after the last spectrum is searched and printed, the focus will be returned to the ChemStation software. If spectra are already in the NIST MS Search Program’s **Spec List** Window, the **Append/Overwrite/Cancel** query will be displayed.

The **Toggle to NIST Library Program** option either starts the NIST MS Search Program or switches to it if it is already running. You can return to the ChemStation software by holding down the <Alt> key and pressing the <Tab> key, or you can select the NIST Program’s **Switch To Caller** Button. You can then return to the NIST MS Search Program by using the <Alt><Tab> command or by selecting the **Toggle** option from the **Options For NIST Library Search** dialog box.

The **Copy ChemStation Lib to NIST Format** option is described in **Appendix 3**.

AUTONIST Macro

This is a non-interactive macro that can be executed using the command line instruction:

MACRO “AUTONIST.MAC”, go

or from within the Method. Every Method directory contains a file DEUSER.MAC. Edit this file to contain the string:

MACRO “AUTONIST.MAC”,go.

This File is in the \SUPPORT.HP directory of the CD ROM and must be copied to the \HPCHEM\MSEXE directory.

When the Method is run, the AUTONIST Macro will execute. The chromatogram is auto-integrated, and the spectra representing the top of each chromatographic peak are stored in a text file. The focus is then switched to the NIST MS Search Program. The spectra are imported into the Program’s **Spec List** Window. If spectra are already in the **Spec List** Window, they will be overwritten.

If **Automatic Search On** and **Return Focus to Caller upon Completion** have been selected in the NIST MS Search Program’s **Automation** dialog box, the spectra will automatically be searched. After the last spectrum is searched, the focus is returned to the HP MS software. In order for this macro to function properly, it will be necessary to have selected the above two options in the **Automation** dialog box.

APPENDIX 3: Copy ChemStation and Other Libraries to NIST User Library Format

This is a separate program called by selecting the **Lib2NIST Converter** icon on the **NIST Mass Spectral Database** program group. This program allows you to select mass spectral libraries in a variety of formats (along with structures or structures in separate files) and copy them to the NIST user library format. For all 32-bit Windows (Windows NT, Windows 98/Me/2000/XP/Vista) platforms, the **Lib2NIST** converter program replaces the **HP2NIST** program that was provided with previous versions of the NIST MS Search Program. The use of the **HP2NIST** program is described at the end of this section. Another feature of the **Lib2NIST** program is the ability to take user libraries that were developed by using versions 1.5 – 1.7 of the NIST MS Search Program and copy them into a format fully compatible with the current version of the NIST MS Search Program.

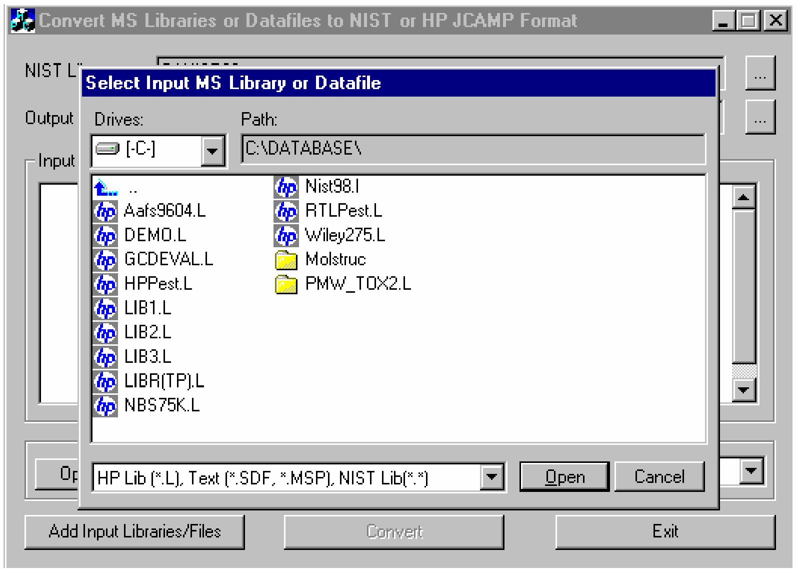

When stated, the **Lib2NIST** converter program displays a file dialog box overlaid on the program's screen.

Opening display of **Lib2NIST** program

Select the library to be copied and then click on the **Open** button. The drop-down arrow will also let you select from a variety of JCAMP formats. More than a single library can be selected in this dialog by using the standard Windows multiple-file-selection techniques.

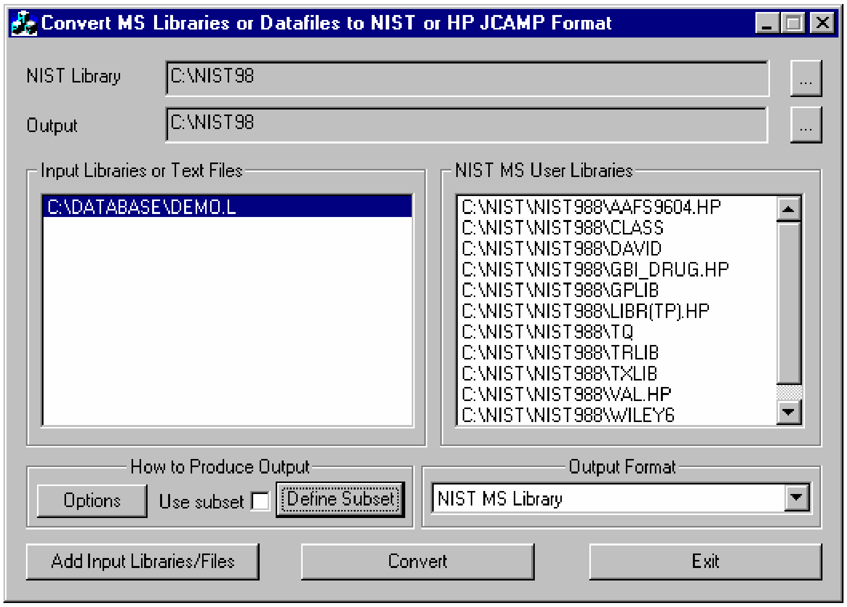

Lib2NIST converter program display

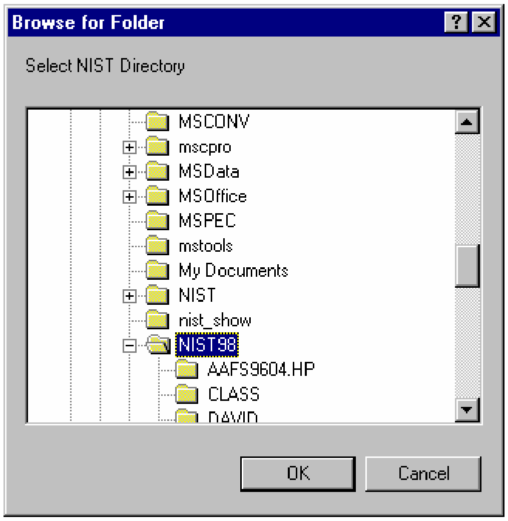

The selected library(ies) will now be listed in the "Input Libraries or Text Files" pane of the program's display.

The **NIST Library** and **Output** locations can be changed selecting the appropriate button located to the right of the two fields. This will result in the display of the **Browse for Folder** dialog box.

Select the desired directory (folder) and then click on the **OK** button to change the location.

The **Define Subset** button will display a dialog box that allows for the choice of a range of input spectra ID or CAS numbers. The "Use subset" *check box* is grayed, unless an entry has been made in the **Define Subset** dialog box.

After making any desired entries in the **Define Subset** dialog box, select the **OK** button to return to the Program's main display.

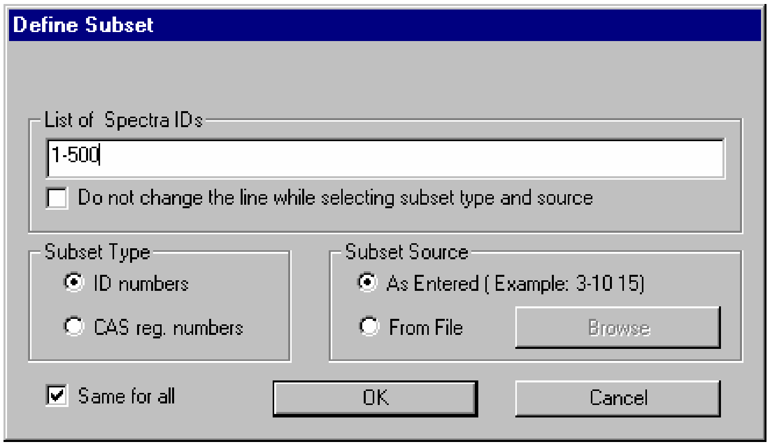

Define Subset dialog box

The **Options** button on the Program's display will display the dialog box shown below. This allows for the entry of mass defect corrections to be applied to *m/z* values of imported spectra and whether or not to: 1) include synonyms from the source file, 2) retain the ID numbers (or sequence numbers) from the source library, 3) calculate molecular weights (nominal mass) from formula, and 4) restrict the output to MS/MS spectra only.

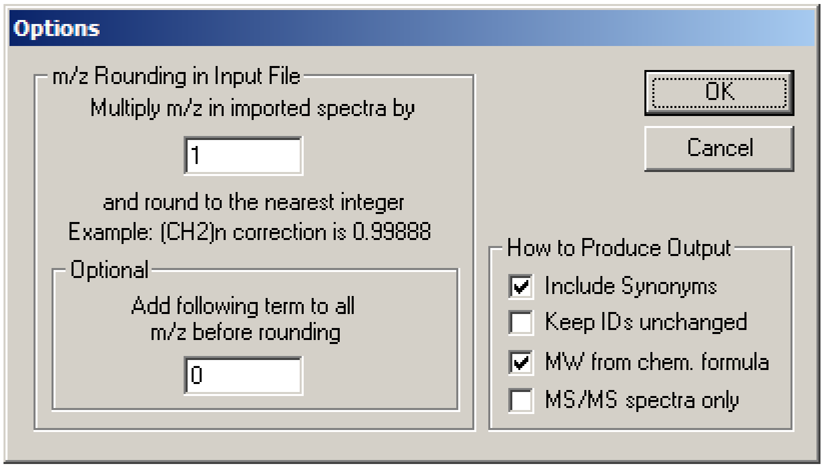

Options dialog box display

Select the libraries to be converted in the "Input Libraries or Text Files" pane of the Program's display, make sure the Output Format is selected correctly, and then click on the **Convert** button. The selected libraries will then be converted and placed in the specified output directory. Advanced features of Lib2NIST are explained in the file CMDLINE.rtf installed with the Lib2NIST. Transliteration rules of extended ASCII characters are listed in the ASCII text file HPTRANS.TBL and may be modified in this file by the user.

APPENDIX 4: Using the NIST MS Search Program with Thermo Fisher Corporation Xcalibur Software

The Thermo Fisher Xcalibur software uses the NIST MS Dynamic Library (dll) as a library search engine. A copy of the NIST MS Search Program and AMDIS may be provided with a copy of the Xcalibur software. The NIST/EPA/NIH Mass Spectral Library may be optionally provided by the Thermo Fisher Corporation.

To add the NIST/EPA/NIH Mass Spectral Library to the Xcalibur software, run the NIST 08 MS Library setup. If it locates the following NIST MS Search Program and the AMDIS directories:

C:\Program Files\NISTMS\MSSEARCH
C:\Program Files\NISTMS\AMDIS32

then let the setup install NIST 08 in these directories. The NIST 08 MS Library will then be properly installed along with any necessary program updates.

More recent versions of the Xcalibur software detect and use NIST MS Search installed in the another directory (for example, c:\nist08\mssearch) and the NIST/EPA/NIH Mass Spectral Library using means explained in the "USE WITH INSTRUMENT DATA SYSTEMS" section of this publication.

Exporting MS/MS spectra to NIST MS Search transfers both the mass spectra the precursor m/z values.

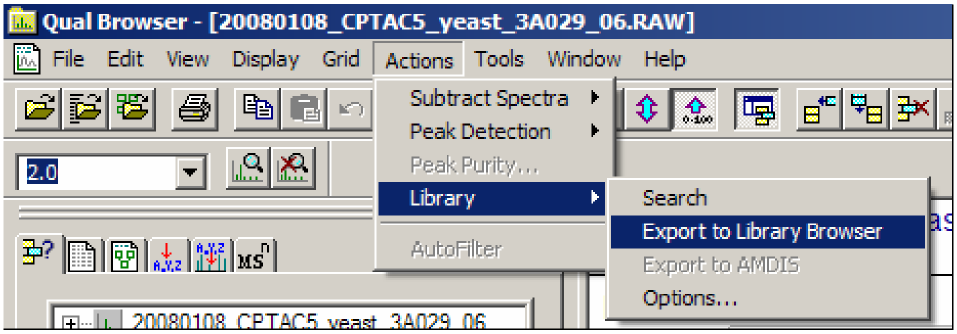

Exporting a spectrum to NIST MS Search

APPENDIX 5: Search Algorithms

There are two general ways that the NIST MS Search Program can retrieve library spectra that resemble the submitted spectrum. These are the "Identity" search and the "Similarity" search. An "Identity" search is designed to find exact matches of the compound that produced the submitted spectrum and therefore presumes that the unknown compound is represented in the reference library. Only experimental variability prevents a perfect match. The "Similarity" search is optimized to find similar compounds and is intended for use when a compound cannot be identified by the "Identity" search (it is probably not in the library).

Screening

For the sole purpose of achieving rapid retrieval rates before actually comparing spectra, modern search algorithms first identify a subset of library spectra with important features in common with the unknown spectrum. While this can vastly reduce search times, it can also screen out the correct spectrum. When this occurs, the correct retrieval cannot appear in the hit list. Because of unavoidable blind spots in simple algorithms, even a closely matching spectrum can be excluded. This is like "throwing out the baby with the bathwater". This is probably the most serious failure of any mass spectral search system.

A variety of filtering algorithms for "Identity" searching were tested in an effort to avoid throwing away correct matches without sacrificing performance. These were tested using the 12,592 spectra that comprised an older version of the NIST/EPA/NIH Selected Replicates Library. The best performing algorithm used a "ranked peaks in common" logic similar to that incorporated in existing data systems. This finds library spectra with the largest number of peaks in common with the unknown spectrum, consistent with a required minimum number of identified spectra. Tests showed this minimum number to be about 50. This procedure retrieved 95% of the matching compounds (5% of matching compounds were lost). By scaling peaks by their *m/z* values, a 98% success rate was achieved at the same search speed. This is the screening logic of the Quick "Identity" search option. By combining results using several screening criteria, a 99.4% success rate was achieved with a modest reduction in search speed. At this level, virtually all correct matches that were screened out were very dissimilar to the unknown spectrum and would have produced low match factors. Both peak scaling and merging of multiple screening results are used in the Normal "Identity" search. A more detailed description follows:

> **Peak Scaling**: The determination of the largest peaks in a spectrum was made after first multiplying the abundance of each peak by the square of its *m/z* value. The most intense peak in the scaled unknown spectrum is compared against the eight most intense peaks in scaled library spectra. The second most intense peak in the scaled unknown spectrum is then compared against the nine most intense peaks in the library spectra. This is repeated in decreasing order of intensity of peaks in the scaled spectrum of the unknown until the eighth most intense peak is compared against the 16 most intense peaks in the scaled library spectra. This is the only screening procedure applied in the Quick "Identity" search.
>
> **Merged Subsets**: Since the use of any single set of peak specifications was found to fail for certain classes of spectra, results of multiple sets of peak specifications were merged to reduce this problem. After extensive optimization studies, four separate peak specifications were selected. The first of these specifications is described above for the Quick search. The others were:
>
> 1) The fourteen largest peaks in the scaled unknown spectrum were matched against the fourteen largest peaks in the scaled library spectra.
>
> 2) The six largest peaks in the original (nonscaled) spectrum were matched against the six largest peaks in the original library spectra.
>
> 3) The five largest peaks along with the "maximum mass" peak in both the unknown and library spectra were matched.

The screening algorithms for the "Similarity" search are similar to the Normal "Identity" search except that scaling and maximum mass peaks are not used. When neutral loss peaks are used in the Hybrid and Neutral Loss search, up to five neutral loss peaks within 64 *m/z* of the molecular ion are used in place of conventional peaks. For neutral loss peaks, abundances in library spectra are required to be within a factor of four of the abundances of corresponding unknown spectra (peak ranking and scaling are not used).

Search

A mass spectrum can be represented as a row vector composed of the ordered peak intensities. It can also be considered to represent a single point in a multidimensional hyperspace defined by the *m/z* variables. Each of the intensities in the row vector represents the value of the coordinate of the spectral point along the individual mass axis in this hyperspace. If two spectra being compared are identical with respect to all the mass intensity pairs,

their point representations in this hyperspace will coincide. If these spectra are very similar, their point representations will be close to one another. The Match Factor, which provides a sense of spectral similarity, may be regarded as the inverse of distance of the two point representations when each spectral vector has unit length.

The dot-product mass spectral search algorithm, which uses the cosine of the angle between the unknown and library spectral vectors, has been optimized by scaling peaks using the square root of their abundance. For the "Identity" search, peaks were weighted by the square of their *m/z* value and a second term was added that compares ratios of adjacent peaks in library and unknown spectra. Its contribution was weighted so that it increased in importance as the proportion of common peaks increases. The only difference in the application of this algorithm to the "Identity" and "Similarity" search is that *m/z* weighting is used only in the former. This defines the search algorithm used for both the "Similarity" and "Identity" searches in the NIST Mass Spectral Search Program.

For additional information, see Stein, S.E. "Optimization and Testing of Mass Spectral Library Search Algorithms for Compound Identification" *J. Am. Soc. Mass Spectrom.* **1994**, *5*, 859–865.

APPENDIX 6: Syntax for Some Constraints in Searches

Syntax of Name Fragments Constraint

Applies to: all searches
Max. length: 39 characters
First release: 2000

This name constraint field can contain up to eight name fragments.
The name fragments are searched in compound main chemical name and in other names (synonyms).

The following examples explain the conditions when the name constraint is satisfied (spaces around && and !! are optional):

"COCA && INE": at least one name contains both "COCA" and "INE"
"COCA && !! INE": at least one name contains "COCA" and doesn't contain "INE"
"COCA !! INE": at least one name contains "COCA"; no name contains "INE"

Syntax of Tags in Comment Constraint

Applies to: all searches
Max. length: 1023 characters
First release: 2006

General

All information in NIST Peptide libraries is represented by the Tag=value convention. Typically, Peptide spectrum comment field may contain following items separated by spaces:

Tag=string
Tag="*string*"
Tag=number

Spaces may be inside a *string* only if the string is in double quotes. In older libraries, an unquoted *string* may also have space(s) inside a pair of parentheses ( ). Spaces are not permitted inside any *Tag* or next to the equal sign.

Comment fields in older libraries may also contain *Tag* not accompanied by the equal sign and *string.*

All *Tag, string, substring*, and *word* comparisons described below **ignore letter case**.

Entering Tags in Comment Constraint

Items in the *Tags in Comment* constraint field should be separated by spaces and/or linefeed characters. Unlike spectra comments, spaces next to equal sign (as well as >, <, >=, <=, and **:**) are allowed inside the items.

Spectra returned must satisfy all entered constraints

Examples

	Item	Comments of the output spectra must have
1	"*consensus*"	A Tag or a space-delimited string *consensus*
2	*parent*	6 letter sequence *parent* anywhere in the Comment
3	*fullname="r.lecvk.c/2"*	Exactly this Tag=string:: *Fullname=R.LECVK.C/2*
4	*fullname=lec*	String following *Fullname*= contains sequence *LEC*
5	*Mods* **:** *ICAT_cl_hi*	String following *Mods*= contains word[1)] *ICAT_cl_hi*
	The following rules apply to strings that are numbers	
6	*Diffmod=6.8*	String following *Diffmod*= begins with number 6.8, for example, *Diffmod=6.8/8* or *Diffmod=6.8*
7	*Diffmod=5.1:6.8*	String following *Diffmod*= begins with number between 5.1 and 6.8, for example, *Diffmod=6.8/8* or *Diffmod=6.8*
8	*Diffmod > 6.8*<br>*Diffmod >= 6.8*<br>*Diffmod < 6.8*<br>*Diffmod <= 6.8*	*Diffmod*= followed by a number greater than 6.8<br>*Diffmod*= followed by a number not less than 6.8<br>*Diffmod*= followed by a number less than 6.8<br>*Diffmod*= followed by a number not greater than 6.8

[1)] *word* is a string delimited by any character but a letter, a digit, or an underscore "_";

If *word* contains spaces or equal sign it should be entered in quotes.

To reverse the meaning of constraints 1-5 (that is, change to "spectra must not have"), enter caret ^ as the first letter:

1	^"*consensus*"	Tag *consensus* is not present
2	^*parent*	Substring *parent* is not present
3	*fullname=*^"*r.lecvk.c/2*"	String tagged *fullname* is not exactly *r.lecvk.c/2*
4	*fullname=^lec*	String tagged *fullname* does not have substring *lec*
5	*Mods* **:** ^*ICAT_cl_hi*	String tagged *Mods* has not word *ICAT_cl_hi*

More formal description

	Item	Comments of the output spectra must have
1	"*Tag*"	Exactly this *Tag* is present in the comment, possibly not followed by the equal sign
2	*substring*	this substring anywhere in the comment
3a	*Tag*="*string* "	exactly this *string* tagged with this *Tag*=
3b	*Tag*=""	This *Tag* has no *string* or its *string* is “”
4	*Tag*=*substring*	this *substring* in the *string* tagged with this *Tag*=
5a	*Tag* **:** *word*	exactly this *word inside string* tagged with this *Tag*=
5b	*Tag* **:** "*word*"	exactly this *word inside string* tagged with this *Tag*=
6	*Tag*=*number*	tagged string = *number* or *string* begins with *number*
7	*Tag*=*number***:***number*	tagged string *number* inside the given range
8a	*Tag* >= *number*	The number in the string less or equal to *number*
8b	*Tag* > *number*	The number in the string less then the *number*
8c	*Tag* <= *number*	The number in the string greater or equal to *number*
8d	*Tag* < *number*	The number in the string greater then the *number*

Notes.

1. Inside an item, spaces are allowed next to signs =, >, <, >=, <=, or **:**.. These signs as well as *Tag* and *string* or *number* or *word* may be located on separate lines.
2. If a *Tag* you created contains characters = or < or > or **:** then use double quotes to search for such a tag, for example, "<*Tag*>"="*string* ".
3. *substring* in rows 2 and 4 may not contain characters < > = **:**.
4. To search for a substring that may be interpreted as a number, for example, 1.23e5, use *Tag*==1.12e5 or *Tag*=="1.12e5" instead of *Tag*=1.12e5
5. To reverse the meaning of constraints 1-2 (that is, change to “spectra must not have”), insert caret ^ in front of the "*Tag*" or *substring*
6. To reverse the meaning of constraints 3-8 (that is, change to “spectra must not have”), insert caret ^ in front of the right hand side expression.
7. To use caret ^ as the first character of a *substring* enter *Tag* == "^*substring*".

Example of a multi-item constraint: The following items

Replicate	- Line 2, substring
RT >= 11.2	- Line 8a, number is greater or equal
Sample = "yeast_Nature_CAM"	- Line 3a, Tag=”string”
Datfile = 011599.dat	- Line 4, Tag=substring

fit the following Comment field:

Replicate RT=11.220m Sample="yeast_Nature_CAM" Datfile="F011599.dat"

More formal description of reversed constraints

	Item	Comments of the output spectra must have
1a	^"*Tag*"	Exactly this *Tag* is not present in the comment
1b	~"*Tag*"	Some of the tags are not exactly this *Tag*
2a	^*substring*	this *substring* is not anywhere in the comment
2b	~*substring*	this *substring* is not anywhere in the comment
3a	^*Tag*="*string*"	all tags but this *Tag* have exactly this *string*
3b	~*Tag*="*string*"	some tags but not this *Tag* have exactly this *string*
3c	^*Tag*=^"*string*"	all tags but this *Tag* haven't exactly this *string*
3d	~*Tag*=^"*string*"	some tags but not this *Tag* haven't exactly this *string*
3e	*Tag*=^"*string*"	this *Tag* has no string or its string differs from *string*
3f	*Tag*=^""	this *Tag* has a non-empty string
3g	**all**=^"*string*"	Each of the tagged strings has not exactly this *string*
3h	**Some**="*string*"	some tagged strings have exactly this *string*

Items 4-8 from the previous section (1.2.2) may be given reversed meaning in the same way as 3a-3f.

Reserved expression all= is same as ^""= and ^=
Reserved expression some= is same as ~""= and ~=

To address a tag named all or some put it in quotes, for example:
"all"=*string* "some"=string
Quoting tags allows searching for exact combination of tags, for example
"Single Tryptic_simple Parent"=358.07 or "Charge=2 Scan"=82

APPENDIX 7: Contributing EI Mass Spectra to the NIST/EPA/NIH Mass Spectral Library

You are invited to submit mass spectra for inclusion in the NIST/EPA/NIH Mass Spectral Library. Descriptions of both ideal and minimum acceptable data are provided below. Please provide data that is as close to the ideal as convenient.

**Characteristic**	**Ideal**	**Minimum Acceptable**
Substance	Pure compounds (single GC component) of confidently known structure that are commercially important, occur in the environment, or are a natural product (including analytically useful derivatives)	Any single chemical compound whose identity is confidently known
Method	Acquired by GC/MS at an ionization energy of 70 eV under conditions such that isotope ratios are not distorted and ions above the molecular ion are absent and processed using AMDIS or other deconvolution software	Acquired at an electron ionization of not less than 20 eV under conditions such that isotope ratios are not grossly distorted and significant ions above the molecular ion are absent; Significant background subtracted
Requested Information:		
Contributor	Contributor's name and/or institution (as you would like it to appear with the spectrum)	Contributor's name and/or institution (as you would like it to appear with the spectrum)
Chemical name	Chemical name (preferably in Chemical Abstracts or IUPAC format)	Chemical name (or structure)
Chemical Abstracts Registry Number	Chemical Abstracts Registry Number	-
Chemical structure	Chemical structure (in MOL/SDF file format)	Chemical structure (or name)
Synonyms	Alternative names (trade names, trivial names, etc.	-
Mass spectrum	Mass spectrum (unit m/z resolution or better) with all observed nominal masses from no higher than m/z 12 to above the molecular ion with intensities of 0.1 % or greater and intensity in electronic format (preferably as an NIST/EPA/NIH user library). Ions at half-mass should be kept in the spectrum and rounded down to the nearest mass.	Mass spectrum (unit mass resolution) with all observed nominal masses beginning no higher than one-third the molecular mass to above the molecular ion with intensities of 2.0 %. Ions at half-mass should be kept in the spectrum and rounded down to the nearest mass.
Instrument type	Instrument type (magnetic sector, linear quadrupole, TOF, etc.)	Instrument type (magnetic sector, linear quadrupole, TOF, etc.)
Instrument model	Instrument manufacturer and model name	-
Ion source temperature	Ion source temperature (°C)	-
Inlet	Inlet (GC, probe, etc.) and inlet temperature (of probe, reservoir, etc.)	-
Retention index	Kovats Retention Index (with the commercial name of the GC phase or, if not available, whether the phase is polar or non-polar)	-
Impurities	Note masses not belonging to the compound and their origin, if known.	Note masses not belonging to the compound, if known.

Submissions and inquiries may be directed to Anzor I. Mikaia or Edward White V, National Institute of Standards and Technology, 100 Bureau Drive Stop 8380, Gaithersburg, MD 20899-8380, USA. E-mail: Anzor Mikaia, anzor.mikaia@nist.gov, Edward White V, edward.white@nist.gov

APPENDIX 8: Support Contacts

In our continuing commitment to quality, the NIST Mass Spectrometry Data Center is always looking to improve the quality of our Mass Spectral Libraries and programs for accessing them. If you have comments or questions about the quality of these or other Standard Reference Databases available from the NIST Standard Reference Data Program, please let us know by contacting:

Joan Sauerwein
National Institute of Standards and Technology
Standard Reference Data
100 Bureau Drive, Stop 2310
Gaithersburg, MD 20899-2310
Internet: srdata@nist.gov
Phone: 1 (301) 975-2208
FAX #: 1 (301) 926-0416
Web site: http://www.nist.gov/srd

If you have questions or problems pertaining to the mass spectral data or use of this program or just want to suggest improvements contact:

Dr. Stephen Stein
National Institute of Standards and Technology
100 Bureau Drive, Stop 8380
Gaithersburg, MD 20899-8380
Internet: stephen.stein@nist.gov
Phone: 1 (301) 975-2505
FAX #: 1 (301) 926-4513

or

O. David Sparkman
5354 Thunderbird Court
Antioch, CA 94531
e-mail: ods@csi.com
Phone: 1 (925) 754-5003
Cell: 1 (209) 483-5740
FAX#: 1 (772) 264-6150

NIST provides updates and enhancements to the NIST Mass Spectral Search Program and AMDIS. These can be downloaded from http://chemdata.nist.gov.

Upgrades to the NIST/EPA/NIH Mass Spectral Database must be purchased.
