# Supplementary material for: Plasma Metabolomics Analysis Based on GC-MS in Infertile Males with Kidney-Yang Deficiency Syndrome
Source: Evid Based Complement Alternat Med. 2017 Sep 25;2017:6270195. doi: 10.1155/2017/6270195 (PMC5674502; doi:10.1155/2017/6270195)
Supplement: Supplementary file 1 — S1 File: Original data of all participants. The rows represent 122 samples and the columns represent 37 metabolites' relative concentrations. S2 File: Ethics Review Table. S3 File: User's Guide of NIST program. S4 File: Tables. [file 6270195.f1.zip › Supplementary Tables (1).pdf]

Supplementary information

Table 1 Qualitative and quantitative analysis of metabolic profiles of healthy control (HC) Group and KYDS Group.

No.	Metabolites	HMDB	Relative concentrations (%)	
			KYDS (n=67)	HC (n=55)
1	Oxalic acid	HMDB02329	0.093±0.023	0.090±0.030
2	Pyruvate	HMDB00243	0.016±0.008	0.018±0.006
3	Lactate	HMDB00190	1.771±0.513	1.853±0.372
4	Alanine	HMDB00161	0.146±0.059	0.147±0.046
5	Phenylpropionic acid	HMDB00764	0.296±0.083	0.257±0.069
6	Glycine	HMDB00123	0.061±0.028	0.068±0.069
7	12-hydroxybutyrate	HMDB00008	0.026±0.008	0.039±0.011
8	N-Acetylglycine	HMDB00532	0.007±0.003	0.005±0.001
9	23-hydroxybutyrate	HMDB00357	0.030±0.022	0.036±0.034
10	α-Hydroxyvaleric acid	HMDB01863	0.010±0.003	0.007±0.003
11	Trisiloxane	NA	0.007±0.004	0.006±0.002
12	L-Valine	HMDB00883	0.101±0.043	0.103±0.050
13	L-isoleucine	HMDB00172	0.035±0.009	0.045±0.025
14	Proline	HMDB00162	0.071±0.035	0.067±0.037
15	Fumaric acid	HMDB00134	0.007±0.003	0.007±0.003
16	L-serine	HMDB00187	0.069±0.009	0.061±0.014
17	Creatinine enol	HMDB00562	0.025±0.008	0.027±0.009
18	2,3,4-Trihydroxybutyrate	HMDB00613	0.010±0.004	0.011±0.005
19	Ornithine	HMDB00214	0.031±0.016	0.038±0.019
20	Glutamic acid	HMDB00148	0.024±0.009	0.030±0.013
21	Phenylalanine	HMDB00159	0.033±0.009	0.038±0.007
22	Lysine	HMDB00182	0.037±0.021	0.048±0.012
23	1,5-Anhydroglucitol	HMDB02712	0.191±0.040	0.122±0.056
24	Fructose I	HMDB01076	0.010±0.015	0.008±0.002
25	Fructose II	HMDB06800	0.012±0.012	0.020±0.006
26	Galactose Glucitol	HMDB05826	0.031±0.009	0.040±0.018
27	Glucose I	HMDB01306	1.953±0.360	1.846±0.417
28	Glucose II	HMDB01401	0.522±0.176	0.504±0.170
29	L-tyrosine	HMDB00158	0.036±0.007	0.037±0.007
30	Palmitic acid	HMDB00220	0.297±0.054	0.286±0.060
31	Myo-inositol	HMDB00211	0.025±0.004	0.026±0.009
32	Uric acid	HMDB00289	0.113±0.046	0.108±0.044
33	Linoleic acid	HMDB00673	0.198±0.043	0.187±0.044
34	Elaidic acid	HMDB00573	0.172±0.057	0.166±0.562
35	Stearic acid	HMDB00827	0.137±0.025	0.127±0.222
36	Arachidonic acid	HMDB01043	0.031±0.010	0.029±0.010

37	Cholesterol	HMDB00067	0.365±0.073	0.351±0.063

Concentrations are presented as mean ± SD by the ratio of its peaks area to that of the internal standard on the same chromatogram.

Table 2 Characteristics of the study population (n=122).

	KYDS Group (n=67)		HC Group (n=55)
	ED (n=24)	SA (n=43)	
Age (years)	28.92±5.21*	29.07±4.92*	30.45±5.56
Weight (kg)	74.27±4.36*	76.93±4.74*	75.38±4.80
The course of infertility (years)	2.81±0.62	2.84±0.39	NA
Sperm concentration (10^6/ml)	89.28±40.79	26.99±40.68#	88.98±75.60
a+b grade sperm rate (%)	62.88±10.22	15.12±16.35#	61.27±14.22
b grade sperm rate (%)	33.28±9.95	4.37±6.16#	42.41±10.89
Normal morphology sperm rate (%)	48.75±39.07	5.75±10.34#	33.98±26.82

*p>0.05 when compared between HC group.

# p<0.05 when compared between HC group.
